# The applicability of forensic time since death estimation methods for buried bodies in advanced decomposition stages

**DOI:** 10.1371/journal.pone.0243395

**Published:** 2020-12-09

**Authors:** Stefan Pittner, Valentina Bugelli, M. Eric Benbow, Bianca Ehrenfellner, Angela Zissler, Carlo P. Campobasso, Roelof-Jan Oostra, Maurice C. G. Aalders, Richard Zehner, Lena Lutz, Fabio C. Monticelli, Christian Staufer, Katharina Helm, Vilma Pinchi, Joseph P. Receveur, Janine Geißenberger, Peter Steinbacher, Jens Amendt

**Affiliations:** 1 Dept. of Forensic Medicine, University of Salzburg, Salzburg, Austria; 2 Dept. of Medicine and Health Sciences, University of Florence, Florence, Italy; 3 Dept. of Entomology, Michigan State University, East Lansing, Michigan, United States of America; 4 Dept. of Osteopathic Medical Specialties, Michigan State University, East Lansing, Michigan, United States of America; 5 Ecology, Evolutionary Biology and Behavior Program, Michigan State University, East Lansing, Michigan, United States of America; 6 Dept. of Biosciences, University of Salzburg, Salzburg, Austria; 7 Dept. of Experimental Medicine, University L. Vanvitelli of Campania, Naples, Italy; 8 Dept. of Medical Biology, Amsterdam UMC – location AMC, University of Amsterdam, Amsterdam, The Netherlands; 9 Dept. of Biomedical Engineering and Physics, Amsterdam UMC – location AMC, University of Amsterdam, Amsterdam, The Netherlands; 10 Institute of Legal Medicine, Goethe-University Frankfurt, Frankfurt, Germany; Free University of Bozen-Bolzano, ITALY

## Abstract

Estimation of the postmortem interval in advanced postmortem stages is a challenging task. Although there are several approaches available for addressing postmortem changes of a (human) body or its environment (ecologically and/or biochemically), most are restricted to specific timeframes and/or individual and environmental conditions. It is well known, for instance, that buried bodies decompose in a remarkably different manner than on the ground surface. However, data on how established methods for PMI estimation perform under these conditions are scarce. It is important to understand whether and how postmortem changes are affected under burial conditions, if corrective factors could be conceived, or if methods have to be excluded for respective cases. We present the first multi-methodological assessment of human postmortem decomposition carried out on buried body donors in Europe, at the Amsterdam Research Initiative for Sub-surface Taphonomy and Anthropology (ARISTA) in the Netherlands. We used a multidisciplinary approach to investigate postmortem changes of morphology, skeletal muscle protein decomposition, presence of insects and other necrophilous animals as well as microbial communities (i.e., microbiomes) from August to November 2018 associated with two complete body exhumations and eight partial exhumations. Our results clearly display the current possibilities and limitations of methods for PMI estimation in buried remains and provide a baseline for future research and application.

## Introduction

Decomposition of a cadaver involves the processes of autolysis, putrefaction and decay [1]. These processes are influenced by several biotic and abiotic factors so that decomposition reflects a dynamic ecological process [2] reflected by relationships among such factors as the individual characteristics of the corpse, weather and climate (e.g., temperature and humidity, soil type, etc.), the substrate, and endogenous and exogenous microbes, insect and vertebrate scavengers [3]. Because of these complex associations, estimation of the postmortem interval (PMI) still remains a challenge in forensic practice. While for the early postmortem period there are some generally accepted methods available (e.g. temperature method [4]), there are no standardized methods for estimating the PMI in the intermediate and late postmortem phases [5]. Yet, forensic entomology is considered one of the most accurate ways to determine a minimal postmortem interval even for some later phases of decomposition [6]. Another widely used rapid and simple approach relies upon the grading of gross postmortem changes [7]. Focusing on the progression of decomposition stages as a function of temperature, researchers provided scoring systems to establish minimum and maximum time intervals for decomposition (e.g. total body score, TBS [8], total decomposition score, TDS [6]) to estimate the time since death. Additional approaches that are useful for PMI estimation in later postmortem phases include the investigation of microbial community succession during decomposition [9, 10] and degradation patterns of (muscle) proteins in relation to PMI and temperature [11].

The study of (human) decomposition is a very longstanding task in forensic science. In the context of PMI estimation, a main source of data comes from cross-sectional case observations of decomposing human bodies; however, such observations are limited in their contributions to theory building [12], mostly because of limited sample size [13]. The use of surrogate models for human decomposition provides the opportunity to provide larger sample sizes and controlled conditions for studies of decomposition and PMI estimates. A majority of taphonomy studies have employed swine as a proxy for human corpses, because of similar body proportions, heat isolation (skin resembles human) and intestinal microbial communities related to omnivory [14]. However, the use of swine is also debated among forensic investigators as recent studies conclude that swine show some differences in decomposition and should be used as a substitute for human subjects with caution [13, 15, 16].

Indeed, the establishment of anthropological research facilities has commenced a new era for decomposition studies. Researchers can seize the opportunity to conduct semi-controlled, longitudinal research studies using replicate human remains with known PMI [17]. Practitioners of numerous disciplines can focus their research on understanding the complexity of decomposition to develop more accurate and precise methods for estimating the PMI [17]. To date there are ten human taphonomy facilities operating worldwide, of which eight are located in the United States and one in East Australia [18]. The first facility in Europe opened in 2018 in the Netherlands. The *Amsterdam Research Initiative for Sub-surface Taphonomy and Anthropology* (ARISTA) comprises a 500 m^2^ burial ground dedicated to study decomposition processes of buried human remains [19].

In the present multidisciplinary study, the decomposition process of two body donors, buried at the ARISTA facility, was monitored over a period of 15 weeks. At predefined time points, the bodies were exhumed, or partially exhumed to assess morphology (TBS, TDS), insect activity, microbial communities and skeletal muscle protein degradation. This study aimed to investigate the applicability and challenges of using a multidisciplinary approach to PMI estimation for advanced decomposition stages in buried human bodies.

## Results

### Environmental conditions

**Fig 1:**
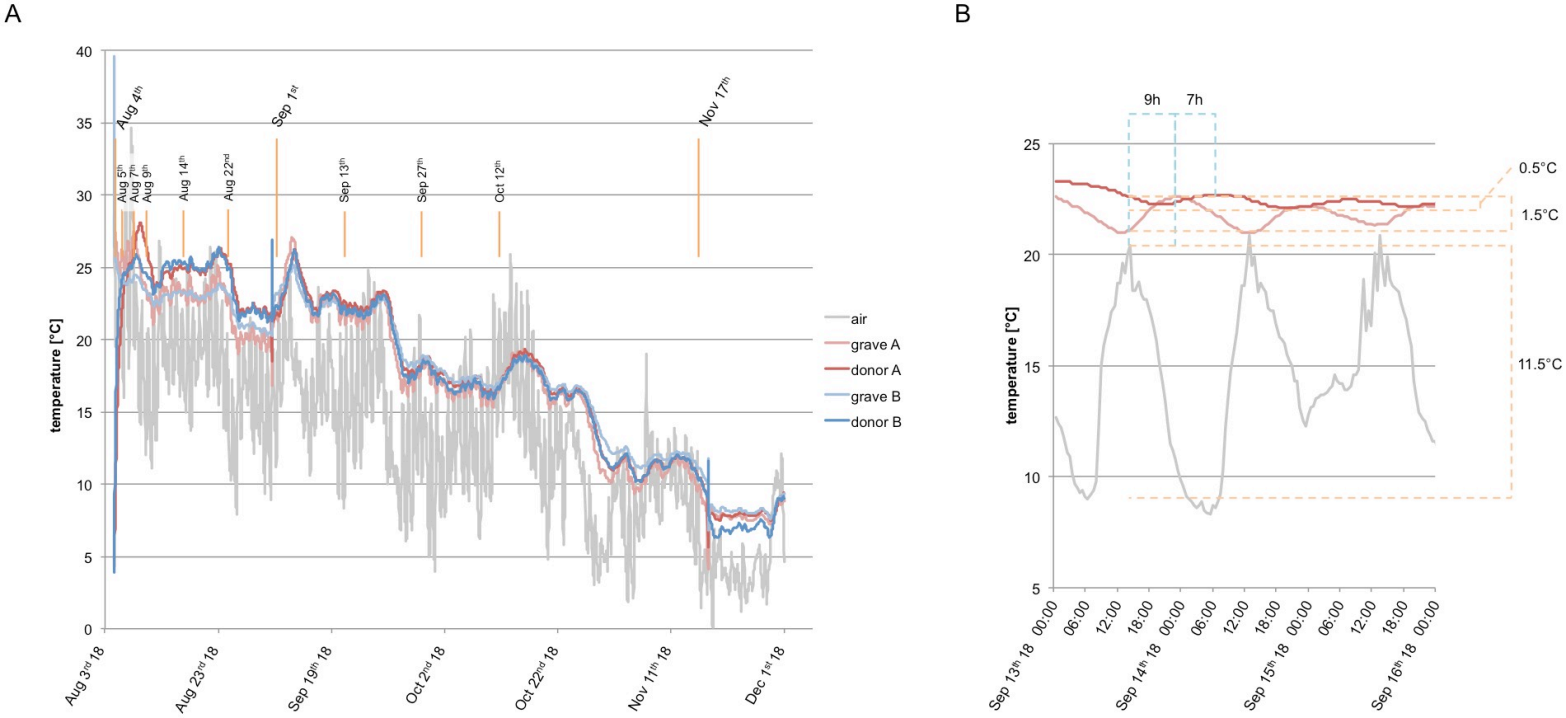
Temperature measurements throughout the experimental trial. (A) Orange lines and dates indicate the time points of the complete body exhumations (long lines) and partial exhumations (short lines). (B) Exemplary section of temperature changes in response to environmental fluctuations. There is a phase shift of approximately 9 h, together with reduced amplitude from air (ΔT_air_ = 11.5°C) to grave (ΔT_grave_ = 1.5°C). A similar effect was observed from grave to body temperature with a phase shift of approximately 7 h and further reduced amplitude (ΔT_donor_ = 0.5°C).

On the day of burial (August 4^th^) air temperature reached 28.5°C. The next three days were hot with a maximum temperature of 34.6°C on August 7^th^. Mean temperatures in all four months were constantly 1–2°C above Amsterdam average, while precipitation was significantly below average throughout the experiment (Table 1). The lowest air temperature of 1.4°C was measured on November 16^th^, the day before the last exhumation. Mean temperatures in the graves, as well as recorded body temperatures exceeded mean air temperature values consistently, but buffered day-night fluctuations. At no time did the soil and the bodies reach sub-zero temperatures, with a minimum of 4.1 and 6.8°C in the graves, and 4.3 and 3.9°C inside the bodies (Fig 1, Table 1).

**Table 1:** Mean temperatures and precipitation throughout each month of the experimental trial.

	air temperature [°C]			precipitation [mm]		grave A [°C]		donor A [°C]		grave B [°C]		donor B [°C]	
	mean	SD	reference*	total	reference*	mean	SD	mean	SD	mean	SD	mean	SD
**August**	18.6	4.2	16.5	52.6	77.0	23.0	2.0	24.0	2.8	22.8	1.3	24.0	2.2
**September**	15.3	4.2	14.3	54.8	79.0	21.2	2.6	21.7	2.2	21.5	1.9	21.5	2.3
**October**	12.5	4.7	10.8	36.6	87.0	16.0	2.2	16.6	1.9	16.8	1.5	16.3	1.8
**November**	7.0	3.4	6.3	23.4	79.0	9.4	1.6	9.7	1.7	10.2	1.8	9.4	2.1

Means and standard deviations (SD) are depicted for air temperature, as measured with the on-site weather station, as well as for temperatures inside the graves and inside the donors, as measured with temperature probes.

*reference climate data (mean temperature) downloaded from www.climate-data.org (March 16^th^ 2020).

**Fig 2:**
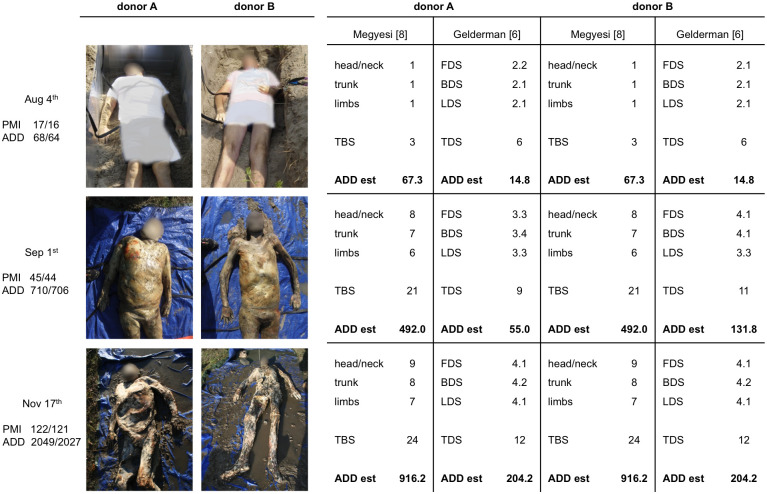
Morphologic scoring according to the keys of Megyesi and Gelderman. Scores were assessed on the day of the burial (Aug 4^th^), and after 28 and 105 days in an earth grave. ADD estimations are based on the formulae published in the original articles. Megyesi et al. [8] reported a Standard Error (SE) of 388.16 ADD for all calculations, Gelderman et al. [6] reported an SE of 52 ADD for outdoor cases. PMI = postmortem interval [days], ADD = accumulated degree days, ADD est = estimated accumulated degree days, TBS = total body score, FDS = facial decomposition score, BDS = body decomposition score, LDS = limbs decomposition score, TDS = total decomposition score.

Prior to the first exhumation, the probes for measuring the temperatures of the graves had been placed too deep, as the body temperature of both donors was almost always above the temperatures in the graves. Consequently, at the second burial (after the first exhumation) the probes were placed at middle depth of the body, as opposed to the bottom of the grave. Thereafter, a double-buffer effect was observed. Environmental temperature fluctuations led to temperature changes with a reduced amplitude and a phase shift in the grave, which again led to smaller and delayed body temperature oscillations (Fig 2b). Data for grave and donor A from September 13^th^ to 15^th^ (e.g.) showed environmental temperature peaks at around 14:30 and fluctuations of approximately 11.5°C. The highest grave temperature peaked at 23:30 with fluctuations of 1.5°C. The body was warmest at 06:30 and only varied by about 0.5°C.

### Decomposition scoring

On the day of the burial (August 4^th^), no visible changes and no skin discoloration were observed except for *livor mortis*, consistent with a very early stage of decay. TBS and TDS assigned were respectively 3 and 6 for donor A and donor B corresponding to 67.3 (TBS) and 14.8 (TDS) estimated ADD. These scores are commonly assigned to bodies at very early stage of decomposition, although 17/16 (in donor A/donor B) days already passed since death corresponding to 68–64 ADD spent by the bodies in coolers at 4°C.

At the first exhumation on September 1^st^, 28 days after burial and 45/44 days after death, the TBS values were 21 for the two donors corresponding to 492 ADD, while the TDS values were different among the bodies. In donor A, TDS 9 was a little bit lower compared with a score of 11 assigned to donor B predicting ADD from 55 to 131.8, respectively. Purging of decomposition fluid out of ears, nose and mouth and brown to black discoloration occurred in donor A. In donor B a slightly advanced stage of decay was observed as represented by caving in of the flesh and tissues of eyes and throat, of the abdominal cavity along with sagging of flesh. ADD predicted by both methods did not correspond to the actual ADD of 710/706.

On November 17^th^, bodies were exhumed more than 3 months after burial (105 days) and 122/121 days after death. Mummification was observed at each body part (head/neck, trunk and limbs) with bone exposure of (way) less than half of the area being scored, and with no disarticulation. Based on the appearance of these late postmortem changes, high TBS and TDS values were assigned to donor A and B, with 24 and 12 respectively, corresponding to 916.2 and 204.2 ADD. Also for this second exhumation ADD predicted by both methods did not correspond to the actual ADDs of 2049/2027.

Hence, all ADD predictions using morphology assessments, 28 and 105 days after the burial did not correspond to the actual values. TBS and TDS largely underestimated the ADD of the burial interval. However, the prediction by TBS was more accurate than TDS (Fig 2). No disagreement between the examiners occurred at any time in the assessment of TBS and TDS.

### Muscle sampling and protein degradation

Partial exhumations and muscle biopsy sampling worked well in the early stages of the experiment. Until day 28 (including the first sample from the left thigh) each biopsy provided a sufficient amount of material, macroscopically easily identifiable as muscle tissue in both donors. Overall, it took about 15 to 20 minutes to complete sampling, specimen storage in the on-site freezer and re-burial. From day 40 onwards, the obtained biopsy material was increasingly difficult to identify as muscle tissue. Additionally, the thighs slipped alongside the femora, making it impossible to target a specific location/muscle. Nevertheless, a sufficient amount of material was collected until day 105, stored and analyzed according to the protocol.

All investigated proteins depicted native bands at the day of burial and underwent qualitative changes during the time course of the experiment. Tropomyosin was present as a characteristic double band (at 38 and 36 kDa) until day 10 in both donors. While bands were lacking in all subsequent donor A samples, the pattern remained detectable on day 18 in donor B. Additionally, a single band was detected on day 28 in the tissue sample collected from the right thigh. Interestingly, no bands were found in the sample collected on the same day from the left thigh, as well as in all subsequent samples.

The native vinculin band (117 kDa) was present until day 3 in both donors and did not appear in any samples with larger PMI. Meta-vinculin (135 kDa) was detected on day 0 in donor A, but in no other sample. A degradation product at 84 kDa was present from day 0 (note that day 0 of the burial does not correspond to a PMI of 0 days) until day 10. A faint band with intensity below 1% of the native band was visible on day 18 in donor B, but no later than that in any of the donors. One sample (donor A, day 3) depicted a degradation product at approximately 75 kDa. From day 3 to day 10 both donors depicted another vinculin degradation product at approximately 63 kDa. While still detectable on day 18 in donor B, in donor A this band was lacking at this time point. The 63 kDa fragment was absent in all subsequent samples.

The native α-actinin band (100 kDa) was present until day 5 in donor A and until day 10 in donor B. A degradation product of 80 kDa appeared on day 3 in both donors and disappeared again on day 18 (A) and day 28 (B). A second degradation product of approximately 65 kDa was detected during the same timeframe in donor B, however only on day 5 in donor A, together with a <1% faint band on day 10.

**Fig 3:**
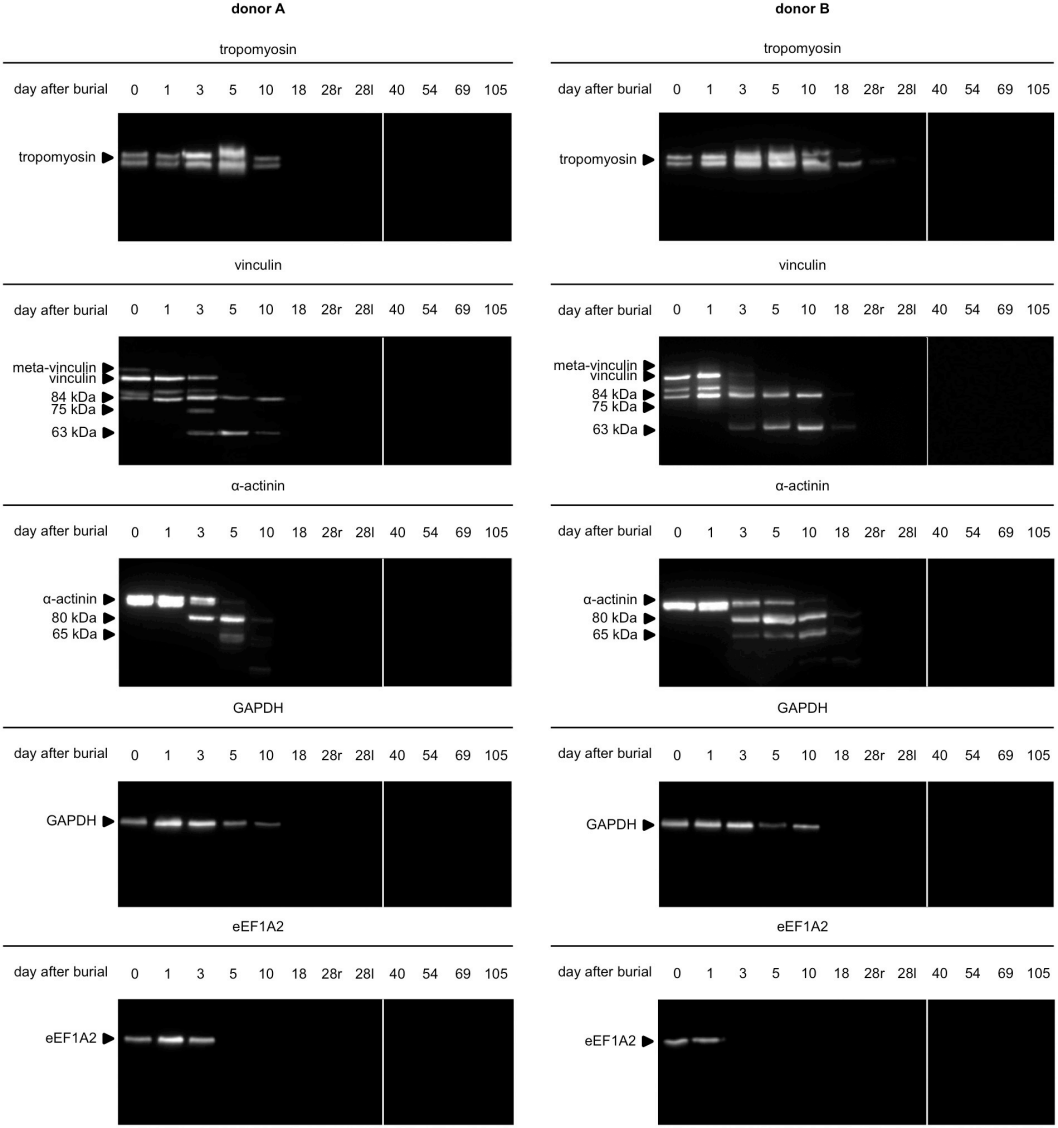
Muscle protein degradation patterns of donor A and donor B. Biopsy samples were taken from the right thigh from day 0 to 28r. After day 28 (first exhumation) the left side was sampled until the second exhumation 28l to 105). The native band of all investigated proteins disappeared at some point during the experiment. Degradation products of vinculin and α-actinin as well disappeared in further consequence. Note that the degradation patterns and temporal coherences are similar but not identical in donor A and donor B. Two gels/blots were required to investigate each timeline (0 to 28 and 40 to 105), denoted by the vertical white lines.

The native GAPDH (40 kDa) vanished after day 10 in both donors. The native eEF1A2 band (50 kDa) disappeared after day 3 in donor A and after day 1 in donor B (Fig 3). Original western blot images are available online (S1 File).

### Activity of insects and other necrophilous species

**Fig 4:**
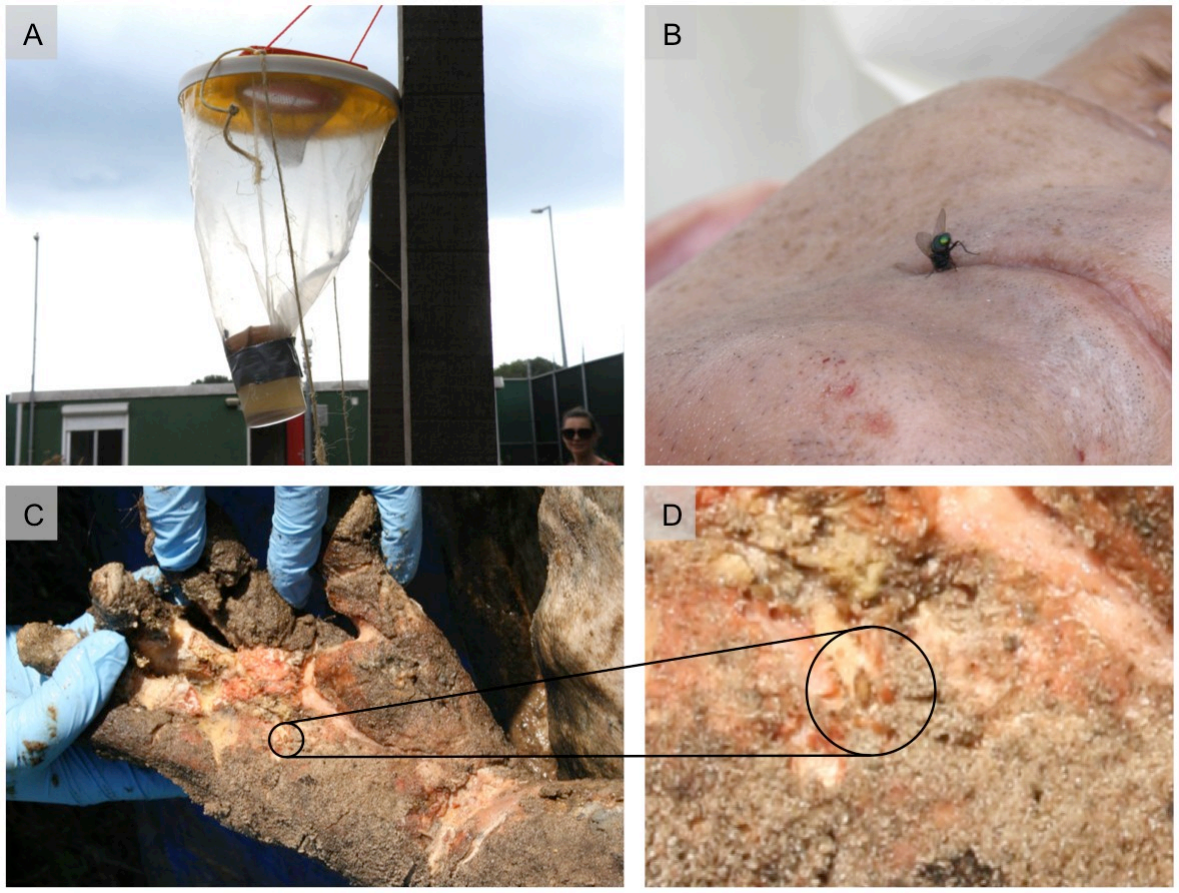
Entomological findings. (A) Day 0 (day of burial), baited flytrap. (B) Day 0, female blow fly *Lucilia sericata* at the mouth corner of donor B. (C-D) Day 105 (second exhumation), a pupa of the scuttle fly *Megaselia scalaris* inside of the right hand of donor B.

Fly activity on the day of burial was moderate, with only a few blow flies of the genera *Calliphora*, *Lucilia* and *Pollenia* caught in the trap, together with 1–2 flesh flies (Sarcophagidae) and house flies (Muscidae) monitored on plants and fences on the site. Only a single female of the species *Lucilia sericata* was found on donor B during two afternoon hours (Fig 4). The fly inspected the oral cavity and nostrils but did not oviposit. The body temperature was below 10°C at this time.

During exhumations, insect activity on the bodies was very low. Donor A showed no infestation during both evaluation days, while Donor B revealed small spots of fly larvae (Day 28 and Day 105) and a few pupae (Day 105) which were distributed in quite different body regions (e.g. right foot and left elbow). The overall number of specimens was below 50, all larvae and pupae belonging to the scuttle fly *Megaselia scalaris* (Diptera: Phoridae) [20, 21].

Additionally, on the left arm of donor B, annelids, initially addressed as Enchytraeids, were collected. Sequence analysis of these specimens produced no exact match in NCBI and in the BOLD databases but has been assigned to the genus *Enchytraeus*. Sequence comparisons with the published barcoding regions of *E*. *moebii*, *E*. *albellus*, *E*. *crypticus*, and *E*. *albidus*, as well as further so far unidentified *Enchytraeus* species from the collection of the Goethe-University Frankfurt, as well assigned the specimens to the genus *Enchytraeus*. A Neighbor Joining analysis with the sequence of *Lumbricus terrestris* was used as outgroup.

### Microbiome

The original sample total intended for sequencing was 48, which included 24 per donor body representing different body sites and three time points (August, September and November 2018). Of the 48 total, 16 samples did not amplify (often because of low DNA concentrations) after extraction and cleanup procedures, leaving a total of 32 samples that were sequenced. Sequencing of the V4 region of the 16S rRNA gene resulted in 2,453,935 total reads, averaging 61,348 (+/- 5,256 SEM) reads per sample. Based on rarefaction curves, using observed sequence variants and Shannon diversity, a rarefaction of 4,000 reads per sample was chosen for additional analyses (S1 Fig). After filtering and rarefaction, 1,558 sequence variants were retained.

#### Overall taxa distribution and changes among body sites and soil communities

**Fig 5:**
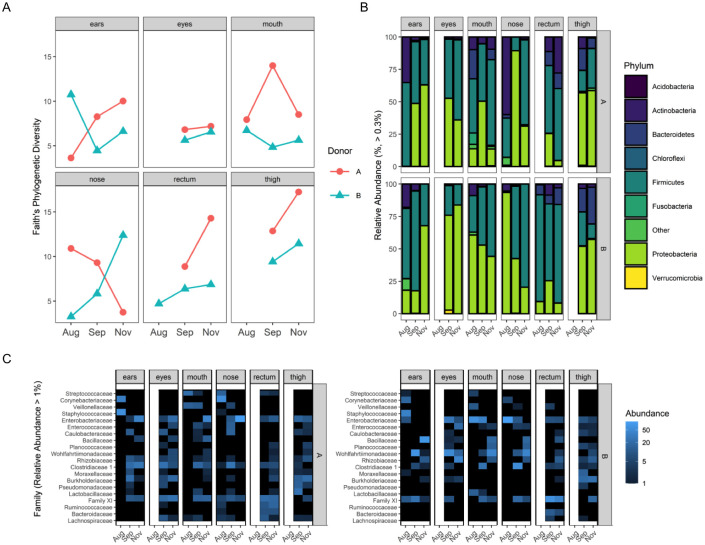
Microbial findings. (A) Faith’s Phylogenetic Diversity. (B) Relative abundance of predominate phyla, and (C) family changes over sampling dates for each body location.

The overall postmortem microbial communities among body sites for the two donors combined was represented by eight phyla, nine families and 27 genera with relative abundances of >0.3%, >3% and >1% among all samples, respectively (S1–S5 Tables). At all levels of taxonomy, there were differential, taxon-specific variations among body sites and over sample dates (Fig 5, S6–S12 Tables and S2–S4 Figs).

At the phylum level, the communities were primarily dominated by Firmicutes and Proteobacteria (Fig 5 and S3 Table), with mean relative abundance of Proteobacteria ranging from 36% in ear communities to 62% in the eyes, and thigh communities >50% (S6 Table). Firmicutes ranged from about 21% in thigh communities to 65% in the rectum, and the other bodies areas 37–52%. In the single soil community, Firmicutes were only 5.7%, while Proteobacteria were the highest (28%) (Fig 5, S5 and S6 Tables). Among the top three phyla, Actinobacteria decreased by 26–31% from August to November, while Proteobacteria and Firmicutes both increased by 1–6% in ear and nose communities (S8 Table). Firmicutes increased and decreased by 7%, in the mouth and rectum communities, respectively. There was little phyletic change from September to November in eye microbiomes. Individual families ranged from 0–40% relative abundance depending on body site and the predominate families generally represented the phylum level changes over time and among body communities (S1 Table). Within any given body site there was not a genus that represented >20% of that community (*Peptoniphilus* was 20% of rectum communities), with the most dominate genera within a body site generally ranging from 5–16% (S7 Table). These results suggest that at genus level and lower, body site communities are highly diverse (S2 and S10 Tables).

#### Microbial community diversity and structure

Microbial diversity depended on body site, donor and sample date (Fig 5), but was not statistically tested because of the limited number of donors (i.e., replication). In general, donor A microbiome diversity was higher than donor B in the mouth on all sample dates, on the first two sample dates for the nose communities and the last two dates for ears and rectum. Nose microbiome diversity increased for donor B over time, while it decreased for donor A. Microbial community structure was not significantly different among sample types present at all dates (PERMANOVA, P > 0.05, S5 Fig and S11 Table), and even when body sites were pooled there still was not a community difference between sampling dates.

Random forest prediction accuracy among mouth, nose and ear microbial communities was poor, with an error rate of 83.33% (Out of Bag Error, OOB) when predicting body site. However, random forest modeling was able to distinguish among sample dates timepoint (mouth, nose, ears, n = 16) with a 5.6% error rate (OOB error, one mouth sample from August 4^th^ was misclassified as November 17^th^) without accounting for differences between donors. The top bacteria genera used in the predictive model (determined by mean decrease accuracy) were mostly bacteria absent on the August 4^th^ sampling but in higher abundances on later dates (S6 Fig). Of the top twenty predictors, thirteen were from the phylum Firmicutes with six representing Proteobacteria and one from Bacteroidetes (S12 Table). Supplemental results are available online (S2 File).

## Discussion

PMI estimation in advanced decomposition stages is an extremely challenging, but important part of a death investigation. Although many techniques have been proposed, most of them are limited to specific time frames or conditions, often the early phases of decomposition. The most promising approach for reliable, accurate and broadly applicable estimations is to have a suite of methods available for making case specific selections that can accommodate the conditions of the remains and changing environmental contexts. The presented study provides the necessary groundwork and important insights into the strengths and limitations of individual approaches, as well as the background for developing multidisciplinary operating procedures, specifically for understanding general applicability of investigated PMI markers (i.e., morphology, protein biochemistry, thanatofauna and microbiome) for bodies in earth graves. Research for this specific purpose is difficult because observations and collections are only possible with disturbance of the body and its environment. With the exception of muscle tissue sampling, which was possible with minor interference, the other assessments required complete body exhumations. Depending on the research question, a subtle balance between least possible disturbance and maximum outcome had to be defined. Similar compromises have to be considered, not only for buried bodies, but when several methods are to be investigated at once in a collaborative manner. Inclusion and exclusion criteria, physical properties of the site/graves (e.g., dry land or river floodplains or wetlands) and the bodies (clothing), sampling/exhumation intervals and resources are among important aspects to work out. Despite the resulting limitations and the small sample size we did, however, collect valuable data important for assessing the potential and limitations for advanced-stage PMI estimation methods for buried human remains.

### Morphology

Since Megyesi’s study in 2005 [8], decomposition can be scored using a point-based system for the main three anatomical body parts (i.e., head and neck, trunk, limbs). This method was validated by several studies [22–24] and recently improved by Gelderman et al. [6] and Moffat et al. [25]. Based on these research studies, decomposition can be best modeled on Accumulated Degree Days (ADD) rather than just PMI [8, 25, 26]. In fact, the accumulation of thermal energy as a function of the PMI can be derived from descriptive Decompositional Scoring Systems [8, 23, 24, 26].

In the assessment of TBS and TDS, no disagreement between the two examiners occurred. Both assessments are practical, simple and user-friendly to efficiently evaluate human decay. According to previous reports [6, 8, 22–26] these methods are useful tools to depict sequential decomposition processes in a more accurate and objective manner, improving the prediction of PMI by ADD. In this field study, however, ADD, predicted by both methods after two exhumations, did not correspond to the actual ADD of the burial intervals (28 and 105 days) neither to the ADD corresponding to the actual PMI (45/44 and 122/121 days respectively for donor A and donor B). A major factor for this inaccuracy may be the low temperatures before and after burial. Bodies were exposed to cold temperatures (approx. 4°C) for the first 17/16 (donor A/donor B) days after death and afterwards were buried at temperatures ranging from 21.2/23°C in August/September to 9.4/16.8°C in October/November. However, temperature is not the only dependent variable for human decomposition. It is well known that the progression of decomposition in an earth grave can be 3 to 8 times slower than surface decomposition depending on the depth of burial, the lack of oxygen and insect activity [27–29]. Additionally, Vass found that Megyesi’s equation did not consider fundamental factors affecting decomposition such as moisture, pH and partial pressure of oxygen that vary with soil composition surrounding buried bodies. Apart from physical factors, seasonal, environmental, geographical and ecological differences lead to inaccurate calculations making scoring scales poor predictors of PMI across these factors [22, 23, 30–33]. This is also the conclusion of a previous study [30] that did not support the proposition of “universality” of the proposed formulae for bodies above ground in aerobic environment and for burial decomposition in anaerobic environment [34]. Therefore, the false perception of accuracy using decomposition scoring systems is mainly attributed to condensing the complexity of integrating all factors affecting human decay (carrion ecology) into a single algorithm, although the peculiar characteristics of the death scene can highly influence scoring [35].

Mummification, as observed in both donors, is a process of preservation and is characterized by the desiccation of remnant tissues. Especially porous and permeable soil, as present in the ARISTA facility, can easily absorb most putrefactive fluids causing a rapid dehydration of soft tissues and leading to mummification rather than decomposition. Previous work showed the weaknesses of scoring scales and regression models developed to predict ADD when mummification and/or irregular decomposition occurs [36]. Vass recommended caution when assessing mummified remains using universal formulae, because of substantial delays in the appearance of late decomposition stages, contributing to error in PMI estimations [34].

So far, the study of decomposition using ADD has been largely investigated in bodies exposed on surfaces [6, 8, 23–25] and bodies in aquatic environment [26, 37]. Yet, a scoring scale for buried bodies have only been tested by Vass for burial decomposition in anaerobic environment [34].

### Proteins

The postmortem degradation of biomolecules has lately become of forensic interest. Among those molecules are DNA (e.g. [38]), RNA (e.g. [39, 40]), lipids (e.g. [41, 42]) and proteins. Significant progress has been achieved in the analysis of postmortem protein decomposition patterns for PMI estimation in recent years (e.g. [43–46]). This interest is especially the case for muscle protein degradation in animal surrogates [47–50] and humans [11, 48]. Nevertheless, for substantial progress towards routine application in this field, there is still a lack of reference data. Animal models can provide important information on the succession and dynamics of protein breakdown under standardized conditions, but conclusions for human application, especially on behalf of timeframes, remains less understood. Establishment of a reference database of autopsy cases is dependent on a substantial number of cases with varying, yet precisely reported PMI, in combination with strict inclusion and exclusion criteria to thoughtfully consider possible influencing factors. This is not foreseeable or controllable and consumes time and resources. Forensic taphonomy facilities, like ARISTA, provide the exclusive opportunity for serial human samplings and thus reliable conditions to describe degradation patterns over time.

The results of our protein analyses can be summarized to three major findings. (i) Decomposition patterns within the first 10 days post burial are familiar to reference data. Notably, the recorded changes were in close temporal resemblance to data, gathered from non-burial experiments in animal models and humans. (ii) Novel degradation patterns, especially the complete loss of protein bands, were detected between 10 and 28 days post burial. (iii) After 28 days, there were no protein profiles detectable using the current standard protocols.

In detail, (i) the presence of tropomyosin and GAPDH as native bands until 10 days post burial confirms results from previous studies in animal models [47, 48,51,52] and humans [11,48]. Similarly, the loss of native vinculin at 5 days post burial, and the appearance of two degradation products at 1 and 3 days respectively, has already been described in animal models [52] and humans [48] in very similar time frames. The loss of eEF1A2 after 3 to 5 days was reported in both animal surrogates and humans [48], similar to what is reported here, suggesting burial has less affect on the degradation of this protein. Protein decomposition is an autolytic process, proceeding in every cell of the body, regardless of the environment except temperature. Skin depicts a strong physical barrier for bacteria and/or water (dehydration) and prevents alterations of the microenvironment in the early stages of decomposition.

To our knowledge, (ii) many of the detected postmortem protein band alterations have not yet been described in forensic context, or at least could not reliably be assigned to a specific PMI or ADD. These alterations include the total loss of the native bands of tropomyosin (after 18/28 days), α-actinin (10/18 days) and GAPDH (18 days), some of which have previously been used as loading controls due to their ubiquitous expression in mammalian body cells [53, 54]. Based on our results, this practice should be questioned for postmortem tissue. Nevertheless, these band alterations should be further investigated on a larger sample set to evaluate their potential as PMI markers.

(iii) 28 days appears to be the maximum limit for (sensible) application of the analysis of muscle protein degradation for PMI estimation in this study/setting. Buried conditions provide a well-protected environment for autolytic processes. In other conditions, such as natural surface environment, this limit does not necessarily have to be reached. Once the skin barrier ruptures, e.g. by insect activity/feeding, tissues can be severely damaged and protein analysis can be complicated, or impossible [52]. This method, thus offers a valuable opportunity for PMI estimation as long as sufficient samples can be collected (which can be highly influenced by other factors).

### Insects and other necrophilous species

In forensic entomology, most cases are concerned with blow flies as they are keen to colonize fresh dead bodies and are very abundant in urban and natural habits. However, concealed and buried remains are difficult or impossible for them to access. Shallow graves are often characterized by a distinguished fauna [55], which varies with the nature and depth of the burial [20]. In this context the entomological findings were expected, because colonization was rather limited and the present insect taxa can be described as specialized colonizers of concealed and buried bodies. Nevertheless, the small number of specimens was surprising, as the burials took place in summer, i.e. during the usually very insect-active period. There were only few flies during the burial of donor A, demonstrated by the small number of specimens in the trap and the apparent lack of insect attraction to the bodies themselves. To increase the opportunity of insect colonization, donor B was placed on the soil surface for about 2 hours, but just one female *Lucilia sericata* appeared at the scene and did not oviposit after detailed examination. The low interest in the bodies may have been due to their low temperatures. Just arriving from the cooler, the body temperatures were still < 10°C, even in the body placed on the sunny surface for two hours.

While blow flies are unable to gain access to a buried or hidden corpse, Phoridae can still enter the smallest of openings. The adult flies burrow through the soil and oviposit on the corpse [56]. The larvae of the scuttle fly *Megaselia scalaris*, the most common and distributed phorid fly, feed on a very broad spectrum of decaying organic materials, and consequently are also common on human bodies. *M*. *scalaris* is usually most frequent in the first wave of insect arrivals but will oviposit at any stage, when given the opportunity [57]. Few larval specimens were found during the first exhumation, but during the second exhumation in November more specimens were collected, including pupae.

Following the survey and summary by Disney [57], temperature dependent development of *M*. *scalaris* is associated with a minimum PMI of about 3 weeks for donor B (based on the finding of pupae during the second exhumation on Nov. 17^th^). This would be an underestimate of about 10 weeks, but compared to morphology and protein analysis, which were not informative after several weeks postmortem, this could be a helpful estimation depending on the case. When focusing on the first exhumation (day 28), the entomological findings were close to the real timeline, as the feeding larvae of *M*. *scalaris* indicated a minimum PMI of about 2 weeks, much closer to the real burial time. However, the present study highlights 3 important challenges of forensic entomology: i) it can (obviously) just be applied when insects colonize the body: while donor B could be analyzed with entomological methods, for donor A there was no entomological evidence despite the fact that the body was buried only a short distances away from donor B. Whether random factors or internal properties of the body were responsible for the lack of insect activity remains unclear. ii) forensic entomology always provides a minimum PMI, which can be identical to the time since death; but when it comes to PMI > 6 weeks this method is limited and much more variable, especially under special circumstances like burials. iii) Many insect species (of potential forensic interest) are still not understood regarding their development and their biology, and there is a need for acquiring reference data.

In addition to entomological specimens several dozen Enchytraeids were collected from donor B. Enchytraeids, also known as potworms, are a widely distributed group of small- to medium-sized earthworm-like animals (Annelida: Clitellata), but we were not able to determine the exact species using the barcode region. Within the genus *Enchtytraeus*, species are extremely difficult to discriminate, and identification by DNA barcoding and database analysis via NCBI or BOLD is only possible for a few taxa, often from a single specimen. In addition, sequences of several unclassified specimens have been uploaded so that overall DNA barcoding is not yet a reliable tool for the identification of enchytraeids at the species level.

So far, information on the effect of carcass decomposition on enchytraeids and vice versa is very limited. Some species commonly occur in large numbers and populations up to several hundred thousand per m^2^ and have a significant effect on processes such as decomposition, especially in soils where earthworms are absent. It has been shown that they accelerate the rate of decomposition of dead plant material by feeding on it directly and also by influencing microorganisms [58], but some enchytraeid species are known to assemble at places with concentrations of dead organic matter of both plant and animal origin [59].

### Microbiome

Similar to other studies examining the microbiome associated with buried cadavers or their surrounding soil [60–62], we were able to provide initial characterizations of the human postmortem microbiome in burial cases. Bacterial communities associated with the buried donors had high levels of variation but went through successional changes. Such community changes may have utility in contributing to a postmortem interval estimate, as has been shown for surface exposed animal surrogate and human remains [63–65]. Unfortunately, we were unable to characterize the surrounding soil in a comprehensive manner, but given the frequency of exhumation disturbance to the soil during burial and re-burial, it is difficult to determine if soil communities would reflect disturbance or both decomposition and disturbance.

Though the microbiomes of the two donors displayed differing trends in microbial diversity, we were able to classify sample date (i.e., a postmortem burial interval) with a high degree of accuracy (only a 5.6% error rate), and identified a number of genera that were important to the classification model. The top ten genus level indicators used in the classification model included a variety of both aerobic and anaerobic bacteria. While some of the genera include taxa which are predominantly associated with human digestive systems (e.g. *Tissierella*) [66], others are associated with environmental habitats (e.g. *Sphingobacterium*) [67], as opportunistic pathogens (e.g. *Brevundimonas*) [68], or are ubiquitous (e.g. *Bacillus*). Our findings were similar to other work on microbial indicators of PMI estimates showing that while microbes can be used as predictors, the taxonomic groups and level which best predicts PMI are dependent on the surrounding environment [64,69]. Though the limited number of donors and time points in this study did not allow for a robust testing of a continuous interval model, in other studies of postmortem microbiomes, data were able to provide a promising tool for PMI investigation in a variety of habitats and conditions [63,65,70,71].

Both soil composition and environmental conditions play a substantial role in determining the structure and abundance of microbes within the soil community [72–74], and would likely influence the colonization of buried cadavers by the surrounding soil. In the pursuit of more robust models with forensic utility (moving beyond predictions at a single site or small geographic range), conducting experiments in a range of both soil conditions and geographic locations, provides opportunities to develop baseline information regarding microbial succession in different environments as well as providing data which may be useful in the validation of future models. This experiment conducted at ARISTA, to our knowledge, is the first description of postmortem microbiomes from buried donor cadavers in central Europe, and we have shown that these communities of microbes have the potential to be useful for models to estimate postmortem burial intervals. However, we also recognize that there were only two donors in this foundational study and there is a need to increase sample sizes of human donors to better account for individual variation in demographics such as body mass, age, sex and circumstances of death that will be important for more robust and generalizable models [64]. Nevertheless, this initial work with a limited sample set demonstrated that both community structure and some individual bacterial taxa could be used to classify sample time point categories (e.g., PMI) with reasonable accuracy; however, there was not sufficient statistical power to perform regression models to better represent a continuous timeline. While we were limited to modeling individual time points and not along a continuum, the descriptive characterizations (e.g., community diversity and taxa relative composition) did show qualitative successional changes over time that could be potentially useful in identifying individual taxa as important to indicators of large differences in time since burial, much like that done recently in an aquatic habitat [75]. Such information has potential forensic utility especially in cases when other forms of evidence (e.g., insects) are not available.

## Conclusion

PMI estimation in buried bodies is a challenging task and available approaches largely suffer from limited reference data. Distinct postmortem changes were observed with every used method in this study. Morphology assessments worked well, using existing reference tables, however, temporal coherences are inadmissible. Correction factors or specific calculation models are conceivable, but require additional research. Muscle protein degradation proceeded in similar patterns compared to data from un-buried corpses and animal models. Even temporal coherences appear likely, which could be explained by a similar degree of protection from exterior influences in buried and un-buried bodies. However, this approach turned out to be clearly restricted to early and intermediate postmortem stages (until approximately 28 days). Ecological markers, such as microbes, insects and other necrophile taxa can provide crucial clues for the estimation of the (minimum) PMI and are yet among the most important traits in forensic practice, especially entomology. However, reference data for buried bodies is scarce. The validity of this study is limited by a small sample size, the particular geographic site, soil composition and burial depth. Nevertheless, opportunities to study human decomposition in different geographic locations and under varying circumstances are limited, and offer exclusive chances to gain scientific knowledge. ARISTA is a unique research facility, providing the infrastructure and technical know-how for future endeavors.

## Material and methods

### Ethical considerations

All experiments were performed in accordance with international and institutional ethics guidelines, in accordance with Dutch legislation and the regulations of the medical ethical committee of the Amsterdam UMC at the location Academic Medical Center.

The Dutch Burial and Cremation Act (‘Wet op de Lijkbezorging’, WLB; Article 1 and Article 67) describes donation to science as one of the three possible final destinations of human remains (the other two being burial and cremation). The procedure of body donation to the Department of Medical Biology of Amsterdam University Medical Center (Amsterdam UMC)- location AMC and the subsequent use of these bodies for scientific research is not subject to medical ethics review.

The Dutch Act on Human Subject Research does not cover research with bodies, donated in the context of the Burial and Cremation Act. Moreover, Dutch legislation does not have other regulations in place that require review in case of concrete research protocols with donated bodies.

The Department of Health Law of the AMC approved the procedure for body donation. The Medical Ethics Committee of the AMC, provided a waiver for individual studies that make use of donated bodies. The Department of Medical Biology, being responsible for the body donation program, reviewed the protocols, methodology, academic merits, eventual privacy and ethical issues, as well as environmental issues.

### Body donors and burial site

Two corpses were obtained through the body donation program of the Department of Medical Biology, Section Clinical Anatomy and Embryology, of the Amsterdam UMC at the location Academic Medical Center in The Netherlands and were buried at the *Amsterdam Research Initiative for Sub-surface Taphonomy and Anthropology* (ARISTA). The two males died little over two weeks before the initial burial (precise dates can not be disclosed, as they can potentially be traced back to the donors) and were stored in body bags in 4°C cooling units. Table 2 provides information on the donors. As stipulated by the Department of Medical Biology of the Amsterdam UMC, detailed physical and medical information cannot be disclosed here to preserve anonymity of the donor but can be inspected upon request.

**Table 2:** Details of the two body donors.

	donor A	donor B
***Sex***	male	male
***Age***	approx, 70	approx. 60
***Stature***	medium height, slight overweight	very tall, normal weight
***cause of death***	cardiac arrest	metastatic malignancy
***Comments***		treated with chemotherapy and antibiotics

**Fig 6:**
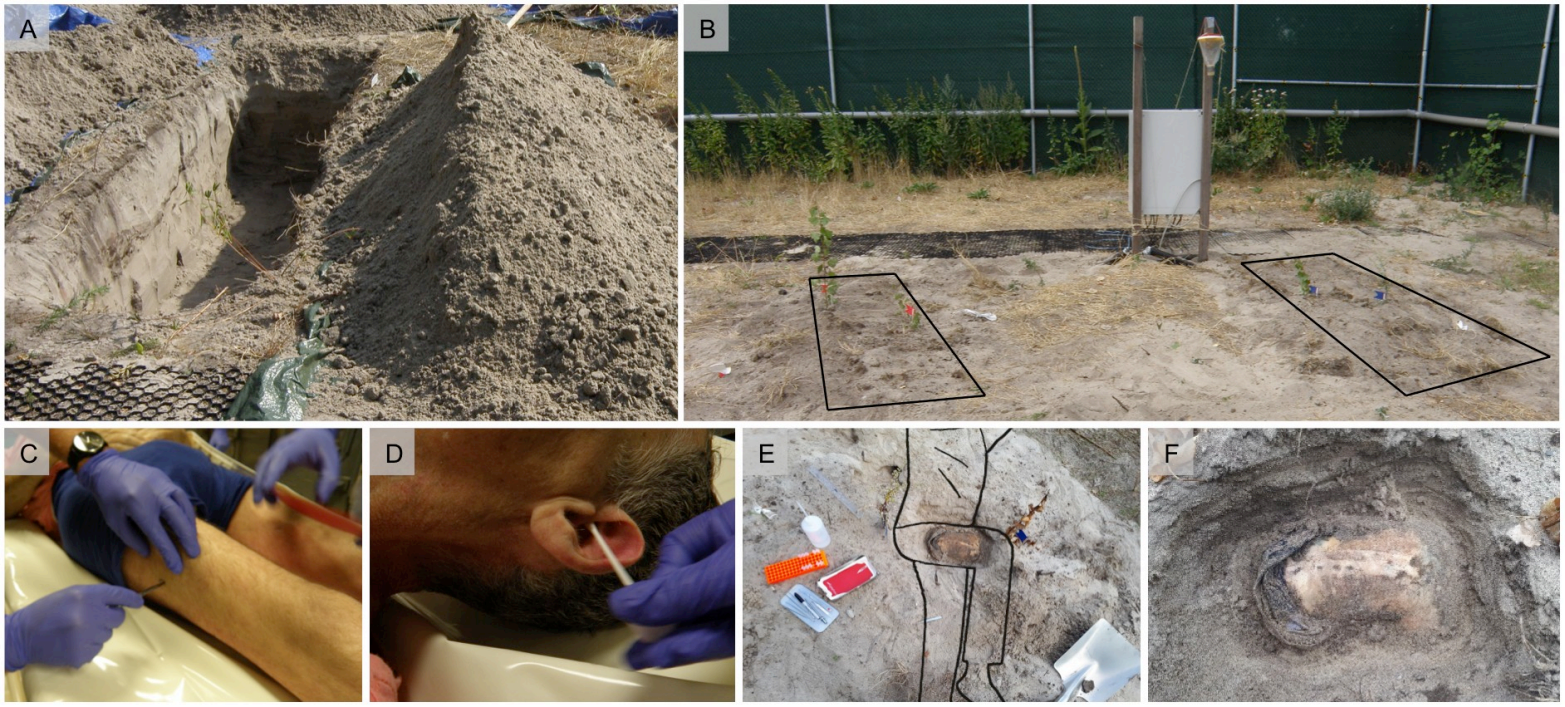
Experimental setup and reference sample collection. (A-B) Earth graves in the sandy soil of ARISTA. (B) Graves of donor A (right, blue flags) and B (left, red flags). In the background: switch box for data acquisition and fly trap (circled in black). (C) Biopsy of the thigh muscle for protein analysis. (D) Microbial swab from the ear. (E) Body position in the earth grave and partial exhumation of the thigh. (F) Detail of a partial exhumation of the thigh with a series of skin openings for muscle tissue biopsies.

On August 4^th^ 2018 two earth graves with a mean length of 2 m, a width of 1 m and a depth of 60 cm were dug into the sandy soil at a distance of 2 m to each other (Fig 6a and 6b). Three layers of soil were separated and placed in different piles to allow for replacement to the original depths during burial. The top 10 cm layer consisted mostly of sand, including some humus and vegetation (roots), loamy sand and minor patches of clay. The two deeper layers (25 cm each) consisted almost exclusively of sand with only slight variations in density and moisture. The graves were equipped with probes for temperature and volumetric water content with 30 min measurements intervals. Additional sensors were placed to recorded precipitation as well as environmental and rectal temperature of the donors. Afterwards, the two donor bodies were removed from the cooling units and respective reference observations and samplings were performed (see below). The bodies were transported to the ARISTA facility. Donor A was buried in the grave assigned to him immediately. Donor B was placed on the surface ground for two hours in cotton pants and t-shirt without coverage to warm up and to be possibly colonized by necrotrophic insects.

The bodies were lowered into the graves using straps and were brought into a lateral position (on their left side), with the right thigh bent at right angle to the body axis. A concrete brick was placed under the right knee to prevent sinking of the leg and/or rotation of the body during the burial and to hinder soil from settling. Starting with the sand from the deepest layers, the graves were carefully re-filled, avoiding the formation of artificial soil cavities (e.g. under the limbs and head). Ultimately, the top layer was replaced on the surface of the grave resulting in a slight elevation (bump) of approximately 10 cm. Indicator flags were placed to mark the position of the hip and the knee. At predefined time points, the bodies were exhumed for subsequent measurements (see below).

### Experimental design

#### Reference observation and sampling

After removing the bodies from cooling units, body scores were assessed according to Megyesi et al. [8] and Gelderman et al [6]. Muscle biopsy samples from the thigh (*M*. *vastus lateralis*) were collected for protein degradation analysis (Fig 6c). Microbial swabs were taken from the eyes, ears, mouth nose, and rectum, as well as the thigh skin (Fig 6d, and see below for details). Reference swabs from the graves were collected from the surface and deeper soil. The local entomology fauna was assessed using an installed flytrap (Fig 1b) and insect nets.

#### Partial exhumations

A total of eight partial exhumations took place on days 1, 3, 5, 10, 18, 40, 54 and 69 after the burial. With increasing time postmortem, the intervals between samplings were extended from 2 to 15 days. For the partial exhumations, soil from in between the two indicator flags (marking hip and knee) was removed with a scoop until thigh skin was visible. Sand residue on the skin was removed using wet cloths. Two centimeters proximal to the previous opening, a small incision was made and a muscle biopsy sample was collected (Fig 6e and 6f).

#### Complete body exhumations

On day 28 and 105 complete body exhumations were performed. Initially, microbial swabs were collected from the surfaces of the graves. Then, soil was removed, again differentiating three layers to retain the vertical structure. Once the upper 25–30 cm of soil had been cleared and the position and composition of the body was determined, additional sand was removed from above and beside the body using small shovels, trowels and brushes. Again, microbial swabs were collected from the skin (thigh) and soil next to the body (i.e. approx. 5 cm beside the thigh). Below the shoulder and hip regions, channels were dug to bring straps into position and lift the bodies out of the graves onto plastic tarps. The clothing was removed, and excess sand was brushed and wiped off again to facilitate photographic documentation and further investigation (see below). When completed (after approximately one hour), the bodies were carefully lowered into the graves and re-buried with soil as described above. After the first exhumation, the bodies were placed on the other (= right) side, with the left thigh in the position described above. This was necessary to provide additional sampling sites for muscle tissue (*M*. *vastus lateralis*) for the following biopsies.

### Assessment of decomposition

#### Morphology

Pre-burial and during the complete body exhumations a total body score (TBS) according to Megyesi et al. [8] and a total decomposition score (TDS) according to Gelderman et al. [6] were assessed independently by at least two authors. Three body regions (i) head and neck, (ii) trunk and (iii) limbs were individually scored to obtain the TBS and TDS. Additional observations, not included in the scoring tables (e.g., discoloration, breakage of skin, insect abundance, ingrowth of vegetation, etc.) were documented and digital photos were taken. Any scoring deviations of the two assessors would have to be additionally documented (written and photographed) and discussed with other authors in order to reach a consensus. Microsoft^®^ Excel^®^ (Ver. 14.5.1) was used for documentation and calculations.

*Muscle sampling and protein analysis*. Tissue samples from *M*. *vastus lateralis* were collected by biopsy from the right (days 0, 1, 3, 5, 10, 18 and 28) and left thigh (days 28, 40, 54, 69, 105). After a punctual incision with a scalpel through the skin, the iliotibial tract and the muscle fascia, a 5 mm diameter biopsy needle was inserted to a depth of approximately 6 cm and muscle tissue was extracted. Approximately 100 μg of tissue material was transferred into a vial tube containing 1 ml of extraction buffer (RIPA buffer (SIGMA) and a protease inhibitor cocktail (ROCHE)). Tubes were stored at -20°C until further processing. After samplings, the openings were sealed with cyanoacrylate glue (Loctite^®^ super glue). The initial sample was collected at one-third the distance from hip to knee along the lateral line of the thigh. Each subsequent sampling was taken 1–2 cm distal from the previous site. All samples were taken from the middle third of the *M*. *vastus lateralis*.

All samples were homogenized in a two-step process. Initially, the tissue material was disrupted using an Ultra Turrax (IKA Werke GmbH & CO. KG). The dispersed samples were treated with high frequency sonication (Hielscher Ultrasonics GmbH) to break up cellular and sub-cellular structures and centrifuged at 1000 × g for 10 minutes. The supernatant was collected and stored at -20°C for further analysis. BCA assay was used to determine total protein concentration, and the samples were diluted to the same level prior to further analysis.

SDS-PAGE were run on 10% polyacrylamide resolving gels and 5% polyacrylamide stacking gels, according to standard protocols [47]. A total of 15–30 μg of protein (depending on the analyzed protein) was prepared, denatured at 90°C for 5 minutes and inserted into the gel wells. Proteins in the gels were transferred onto polyvinylidene flouride (PVDF) membranes (ROTI^®^PVDF, Carl Roth GmbH) and stored at -20°C. Membranes were blocked in blocking buffer (Phosphate-buffered saline with TWEEN and 1% BSA as blocking agent), and subsequently incubated with primary- and secondary antibodies. Between each incubation step, the membranes were rinsed and washed (3 × 10 min) in washing buffer (Phosphate-buffered saline with TWEEN). Primary antisera against the following proteins were used: tropomyosin, vinculin, α-actinin, GAPDH and eEF1A2. HRP-conjugated polyclonal goat anti-mouse, or goat anti-rabbit immunoglobulins were used as secondary antiserum. Staining was visualized by adding chemiluminescence substrate (ROTI^®^Lumin plus, Carl Roth GmbH) and documented with a digital gel analysis system (Fusion FX7, PEQLAB Biotechnology). Protein band intensities were measured using ImageJ software (ImageJ 1.45s, Java 1.6.0_20). Alterations, such as the disappearance of a native band or appearance of additional bands, were considered degradation events. Signals < 1% the intensity of the native bands were considered background and thus no band.

#### Insects and other necrophilous taxa

A modified original Red Top ^®^ Flycatchers (3 l, Ashmoat Ltd., Suffolk) flytrap was placed in the facility on the day of burial to obtain a baseline characterization of insect activity. In addition, one of the two bodies (donor B) was kept on the surface for additional 2 hours, freely accessible to insects. Activities of insects were recorded prior to the burial.

On day 28 and 105, specimens of insects and other necrophilous taxa were manually taken during body exhumation. Soil was removed (see above *complete body exhumations*) and the soil itself, as well as the bodies were visually checked for insects, and other biological activity. If present, specimens were transferred directly into 70% ethanol for killing and storage. Colonization on the surface, e.g. by blow flies, was prevented manually by permanent monitoring. After an initial morphological sorting and classification, genomic DNA from two specimens, which could morphologically be identified as enchytraeids was extracted from the whole individual using a slightly modified phenol–chloroform extraction [76]. Subsequent to ethanol precipitation, a final elution in 50 μl of distilled water was performed and extracts were stored at 4°C until PCR processing. Amplification of the COI barcoding region was performed using the primers LCO1490 (5′- GGTCAACAAATCATAAAGATATTGG-3′) and HCO2198 (5′-TAAACTTCAGGGT GACCAAAAAATCA-3′) [77]. Amplification was performed in a total reaction volume of 25 μl containing 1 unit/μl of Taq DNA polymerase, 2 mM of each dNTP, 8 mg/ml of BSA and 5 pmol of each primer. 5 μl of the DNA extracts were used as template. All PCR amplifications were performed according to Boehme et al. 2010 [78]. PCR products were directly sequenced in both directions using the BigDye^®^ Terminator v3.1 Cycle Sequencing Kit (Applied Biosystems). The protocol included a total reaction volume of 20 μl consisting 3 μl Big Dye, 2.5 μl 5× sequencing buffer, 5 pmol primer and 1 μl PCR product. Protocol for sequencing reaction was 28 cycles of 96°C for 10 s, 50°C for 5 s, and 55°C for 4 min. Sequencing products were purified using gel-filtrated columns (Qiagen, DyeEx 2.0 Spin Kit) and run on an ABI3130 genetic analyzer (Applied Biosystems). Sequence data for forward and reverse DNA strands were edited and aligned manually using the software CodonCode aligner (Version 5.1.5). Sequences were blasted to NCBI reference sequences and afterwards implemented in an NJ analysis and compared with the sequences of specimens of different Enchytraeidae using MEGA (Version 7.0.26).

#### Microbial swabbing, DNA isolation and analysis

Forensic grade DNA free cotton swabs (SARSTEDT AG, Germany) were used to sample microbial communities from different body sites and soil. The swabs were firmly scrubbed against the respective target areas for approximately 10 seconds. On the day of the burial, as well as the two complete body exhumations (day 28 and day 105), the following body sites were swabbed: eyes, ears, nose, mouth, thigh skin and rectum. The entire accessible surfaces (e.g. ears: both ears, outer lobe and auditory canal; mouth: inner lips and cheeks, teeth, all sides of the tongue and gum) were swabbed while twisting the cotton tip to maximize the contact area. Avoiding contact to any other surfaces or aerosols the tips were inserted into test tubes, containing 50 μl of 100% ethanol, snapped off and stored in the on-site –20°C cooling units until further processing.

For DNA isolation and downstream analyses, swabs were processed using previously published protocols [79]. Briefly, the PowerSoil kit (Qiagen^®^) was used for DNA isolation, with the addition of lysozyme (15 mg ml^-1^, Invitrogen) during the lysis step. Once isolated, DNA was quantified fluorometrically using a Qubit 2.0 (Grand Island, NY, USA) and a dsDNA High Sensitivity Assay Kit (Invitrogen). All DNA preparations were then stored at -20°C until further processing. Library preparation and sequencing (2 x 250 bp paired-end reads) were performed using an Illumina MiSeq platform, following previously described methods [80]. To profile the microbiomes for each swab, the variable region 4 (V4) of the 16S rRNA gene was amplified using indexed primers 515f and 806r (5′- GTGCCAGCMGCCGCGGTAA -3′, 5′- GGACTACHVGGGTWTCTAAT -3′) as previously described [80–82]. Demultiplexing and base calling were performed using Bcl2fastq (v 2.19.1, Illumina) and RTA (v 1.18.54, Illumina).

Using default settings in QIIME 2 (v 2020.2) raw sequencing reads were quality filtered [83], while DADA2 and QIIME 2 were used to filter samples, remove low quality reads, singletons, and chimeric sequences [84]. A Naïve Bayes classifier was trained using the region amplified by the primers (515f, 806r, 250 bp) and the SILVA database (v 132) at a 99% confidence level to classify reads before taxonomic assignment using default settings in QIIME 2 [85]. Reads mapped to mitochondria or chloroplast were removed. A rooted phylogenetic tree, created using QIIME2, FastTree (v 2) [86], and MAFFT (v 7) [87] was used to calculate Faith’s phylogenetic distance (Faith’s PD), a common microbial community diversity metric, in addition to Shannon diversity of each sample [88].

Sequencing data were deposited in the National Center for Biotechnology Information (NCBI) Sequence Read Archive (SRA) under the accession code PRJNA643564. Differences in multivariate dispersions between/among groups (i.e., body sites or sample dates) were tested using PERmutational Multivariate Analysis Of Variance (PERMANOVA) and weighted UniFrac distance implemented using vegan package v 2.5–4 [89]. Differences in beta diversity were visualized using Principle Coordinate Analysis (PCoA) plots. To test the utility of using microbial communities to predict time since burial, random forest modeling (RandomForest v. 4.6–14) was used to determine the accuracy of predicting sample timepoint based on microbial community data. Only sample types present on all three microbial sample dates (mouth, nose, and ears; n = 18) (Aug 4th, Sep 1st and Nov 17th) were included in the models. Only categorical modeling (predicting what date a sample came from) was used, as there were too few samples for a reliable regression model. Data were visualized using a combination of ggplot2, ggpubr, and phyloseq packages [90–92] with all code used in analysis available online (https://github.com/BenbowLab/ARISTAproject).

## Supporting information

S1 Raw images Raw western blot images. (PDF)

S1 File Supplemental results microbiome. (PDF)

S2 File (PDF)

S1 Fig Rarefaction curves using observed sequence variant depth and Shannon diversity. (TIF)

S2 Fig Mean relative abundance of predominate families over sampling dates for each body location for both donors (A above, B below). Families shown were represented by at least 3% among samples.
(TIF)

S3 Fig Heat maps showing the relative abundance of bacterial phyla over sampling date and each body location for the two donors (A above, B below). Phyla shown were represented by at least 0.3% among samples.
(TIF)

S4 Fig Heat maps showing the relative abundance of bacterial families over sampling date and each body location (with left and right thigh separated) for the two donors (A above, B below). Families shown were represented by at least 0.3% among samples.
(TIF)

S5 Fig PCoA ordination of bacterial communities among body locations and sampling time points (donor samples pooled). (TIF)

S6 Fig Mean relative abundance of predominate genera identified as indicator taxa in random forest models over sampling dates for each body location (ears, mouth nose) and both donors (A above, B below). (TIF)

S1 Table Family level mean (SD, SE) relative abundances for each sample type of taxa that were >3% in relative abundance across all samples. Mean values are represented by shades of color, with darker indicating more relative abundance among the taxa within a body site or soil.
(XLSX)

S2 Table Genera with a mean absolute >/ = 5.0% change over at least two sample dates for each body site. Samples from donors were pooled for the averages. Blue shaded cells represent positive change, while pink represent negative change.
(XLSX)

S3 Table Phylum level mean (SD, SE) relative abundances for taxa that were >0.3% among all samples. Taxa are arranged from greatest to lowest relative abundance.
(XLSX)

S4 Table Family level mean (SD, SE) relative abundances for taxa that were >3% among all samples. Taxa are arranged from greatest to lowest relative abundance.
(XLSX)

S5 Table Genus level mean (SD, SE) relative abundances for taxa that were >3% among all samples. Taxa are arranged from greatest to lowest relative abundance.
(XLSX)

S6 Table Phylum level mean (SD, SE) relative abundances for each sample type of taxa that were >0.3% in relative abundance across all samples. Mean values are represented by shades of color, with darker indicating more relative abundance among the taxa within a body site or soil.
(XLSX)

S7 Table Genus level mean (SD, SE) relative abundances for each sample type of taxa that were >1% in relative abundance across all samples. Mean values are represented by shades of color, with darker indicating more relative abundance among the taxa within a body site or soil.
(XLSX)

S8 Table Phylum level mean (SD, SE) relative abundances for each sample type (donors merged) of taxa that were >0.3% in relative abundance across all samples. Mean values are represented by shades of color, with darker blue and darker red indicating more or less relative abundance among the taxa within a body site, respectively. The mean percentage change between the first and last sampling dates is given, with taxa bolded that showed >/ = 5% absolute change (for those body sites with multiple sample dates).
(XLSX)

S9 Table Family level mean (SD, SE) relative abundances for each sample type (donors merged) of taxa that were >3.0% in relative abundance across all samples. Mean values are represented by shades of color, with darker blue and darker red indicating more or less relative abundance among the taxa within a body site, respectively. The mean percentage change between the first and last sampling dates is given, with taxa bolded that showed >/ = 5% absolute change (for those body sites with multiple sample dates).
(XLSX)

S10 Table Genus level mean (SD, SE) relative abundances for each sample type (donors merged) of taxa that were >1.0% in relative abundance across all samples. Mean values are represented by shades of color, with darker blue and darker red indicating more or less relative abundance among the taxa within a body site, respectively. The mean percentage change between the first and last sampling dates is given, with taxa bolded that showed >/ = 5% absolute change (for those body sites with multiple sample dates).
(XLSX)

S11 Table Community structure difference tests (PERMANOVA) for: A) among body sites; B) between sample dates, soil excluded; C) between sample dates, with soil included. (XLSX)

S12 Table Top 20 genus level predictors for modeling sample timepoint. Taxa were chosen based on the effect their removal from the random forest model had on overall model accuracy (Mean Decrease Accuracy).
(XLSX)
