# Supplementary material for: The applicability of forensic time since death estimation methods for buried bodies in advanced decomposition stages
Source: PLoS One. 2020 Dec 9;15(12):e0243395. doi: 10.1371/journal.pone.0243395 (PMC7725292; doi:10.1371/journal.pone.0243395)

## tropomyosin

1 AB: anti-tropomyosin
(DSHB CH1-s)
1:500

2 AB: goat anti-mouse/HRP
(DAKO P0447)
1:10000

ThermoFisher iBright
auto exposure
(10-40 sec.)

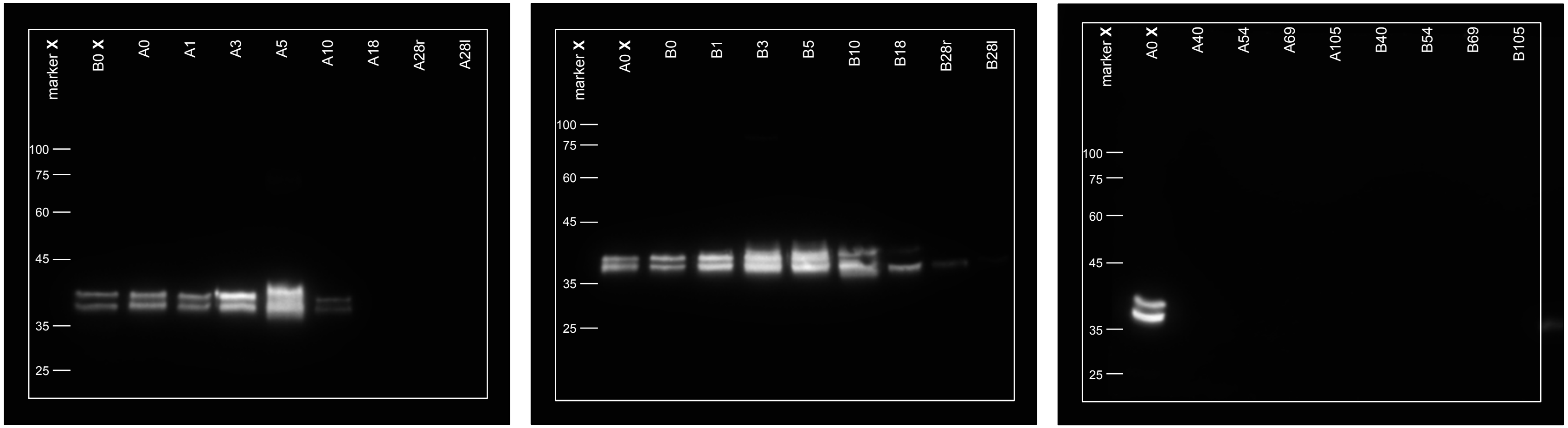


## vinculin

1 AB: anti-vinculin
(SantaCruz 7F9)
1:1000

2 AB: goat anti-mouse/HRP
(DAKO P0447)
1:10000

ThermoFisher iBright
auto exposure
(5-35 sec.)

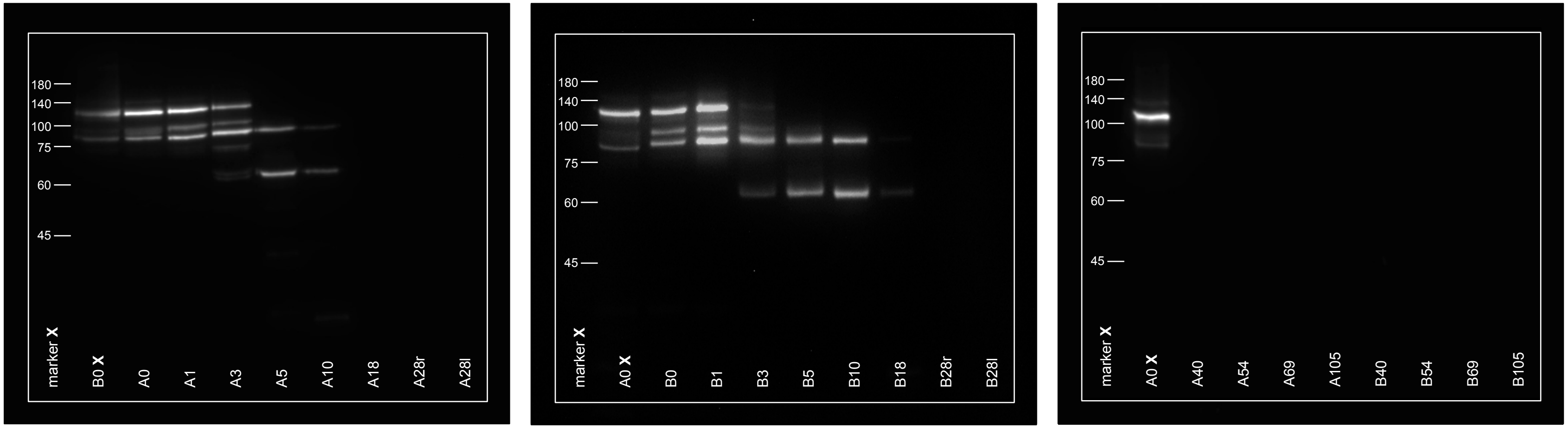

α-actinin

1 AB: anti-α-actinin
(SantaCruz H-2)
1:1000

2 AB: goat anti-mouse/HRP
(DAKO P0447)
1:10000

ThermoFisher iBright
auto exposure
(30-90 sec.)

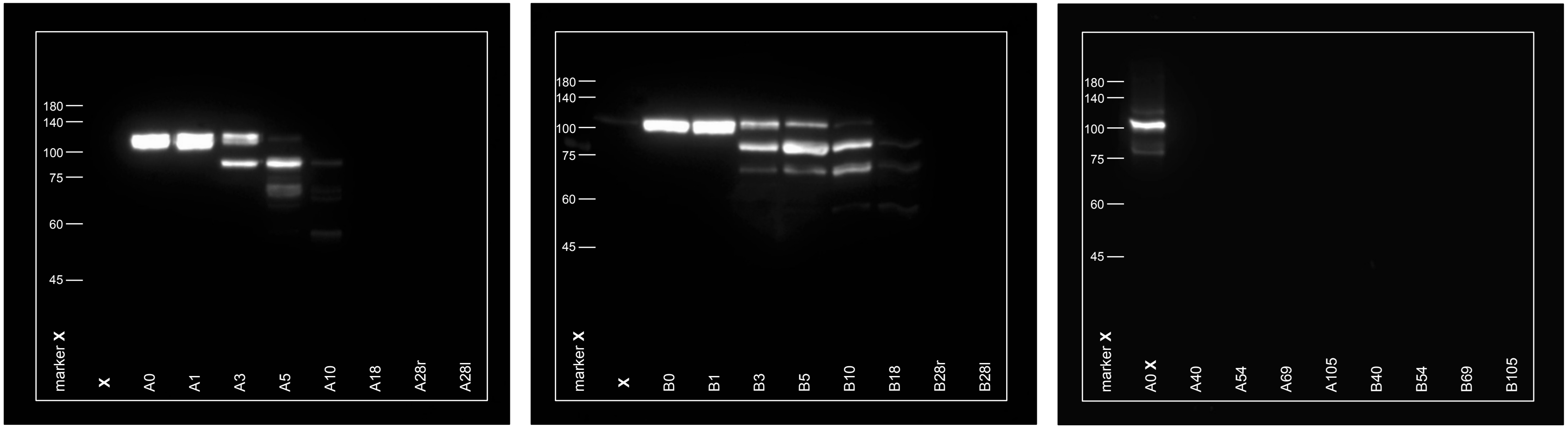


GAPDH

1 AB: anti-GAPDH
(SantaCruz 6C5)
1:1500

2 AB: goat anti-mouse/HRP
(DAKO P0447)
1:10000

ThermoFisher iBright
auto exposure
(5-25 sec.)

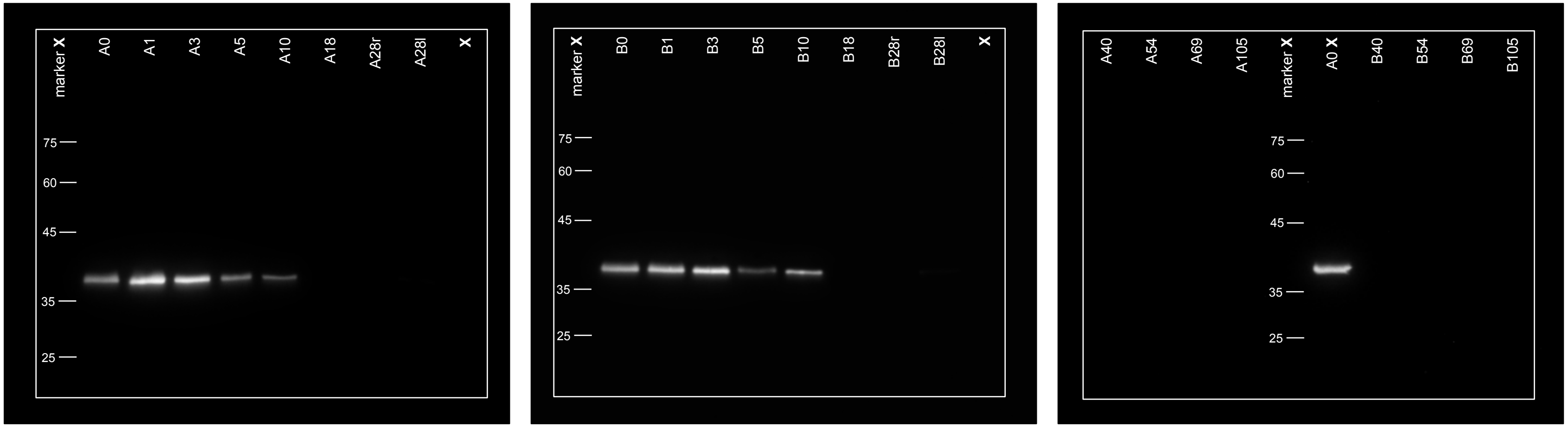

<u>eEF1A2</u>

1 AB: anti-eEF1A2
(Abcam ab153714)
1:1000

2 AB: goat anti-rabbit/HRP
(DAKO P0448)
1:10000

ThermoFisher iBright
auto exposure
(20-40 sec.)

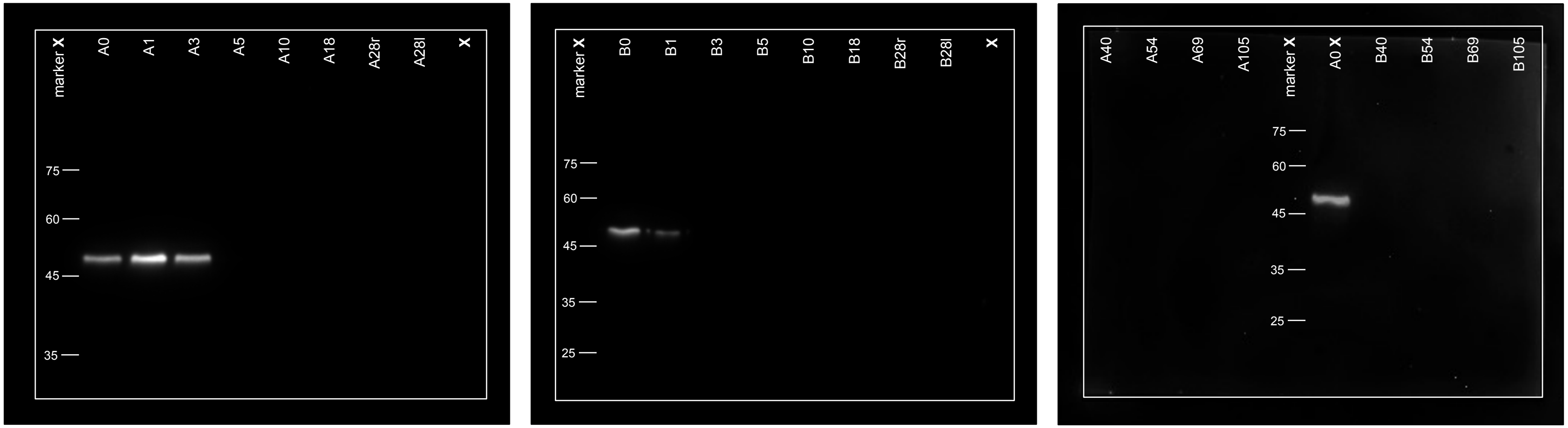

Supplement: S1 Raw images — (PDF) [file pone.0243395.s001.pdf]
