# Supplementary material for: The applicability of forensic time since death estimation methods for buried bodies in advanced decomposition stages
Source: PLoS One. 2020 Dec 9;15(12):e0243395. doi: 10.1371/journal.pone.0243395 (PMC7725292; doi:10.1371/journal.pone.0243395)
Supplement: S1 File — (PDF) [file pone.0243395.s002.pdf]

Supplemental Results

Microbial Communities at the Family Level

The Proteobacteria family Burkholderiaceae made up the highest relative abundance in thigh communities, while the other dominate family of this phylum was Wohfahrtiimonadaceae >15% in both ear and eye communities. Firmicutes was most represented by Staphylococcaceae in ears, while Enterobacteriaceae ranged from 15-36% in eye, mouth and nose communities and Family XI was 40% in the rectum and from 8-13% among most other body sites. All of these top families made up very low relative abundances in soil communities, with no family >1.3%. Within the ears, mouth and nose microbiomes, several families increased by > 10% from August to November: Bacillaceae (ears 38%, mouth 15%); Clostridiaceae 1 (ears 14%, nose 12%); Enterobacteriaceae (ears 15%); Wohlfahrtiimonadaceae (mouth 23%); Family XI (nose 11%) (S9 Table). Those that decreased by >10% in the same communities were Corynebacteriaceae (ears 26%, nose 31%); Staphylococcaceae (ears 49%); but in rectum communities Family XI decreased by 36%. In the eye microbiomes, the Rhizobiaceae increased by 22%, while Wohlfahrtiimonadaceae decreased 15% from September to November (S9 Table).

Microbial Communities at the Genus Level

Ignatzschineria ranged from 8-11% among ear, eye and mouth communities, but was lower in nose, rectum and thigh communities. Providencia represented from 2.7-15% within all body sites except the rectum and ear communities, while Anaerosalibactera was consistently from about 4-6% among all body sites, except the right thigh and rectum. Soil communities were represented by very low relative abundances of most genera, with Bacillus (0.87%) and Providencia (0.80%) being the most predominate. The genera that changed by ≥5% over sample dates varied by body site, with Bacillus increasing by 38% and Staphylococcus and Turicella decreasing by 49% and 20%, respectively, in ear communities (S2 and S10 Table). Providencia and Ignatzschineria (an insect associated genus) were the only genera that changed among three body sites, with Providencia increasing by 7%, 20% and 46% in the eyes, mouth and nose, respectively; while Ignatzschineria decreased by 8% in eye communities, but increased by 22% and 7% in mouth and nose microbiomes, respectively (S2 Table).
