# Supplementary figures and images for: The applicability of forensic time since death estimation methods for buried bodies in advanced decomposition stages
Source: PLoS One. 2020 Dec 9;15(12):e0243395. doi: 10.1371/journal.pone.0243395 (PMC7725292; doi:10.1371/journal.pone.0243395)

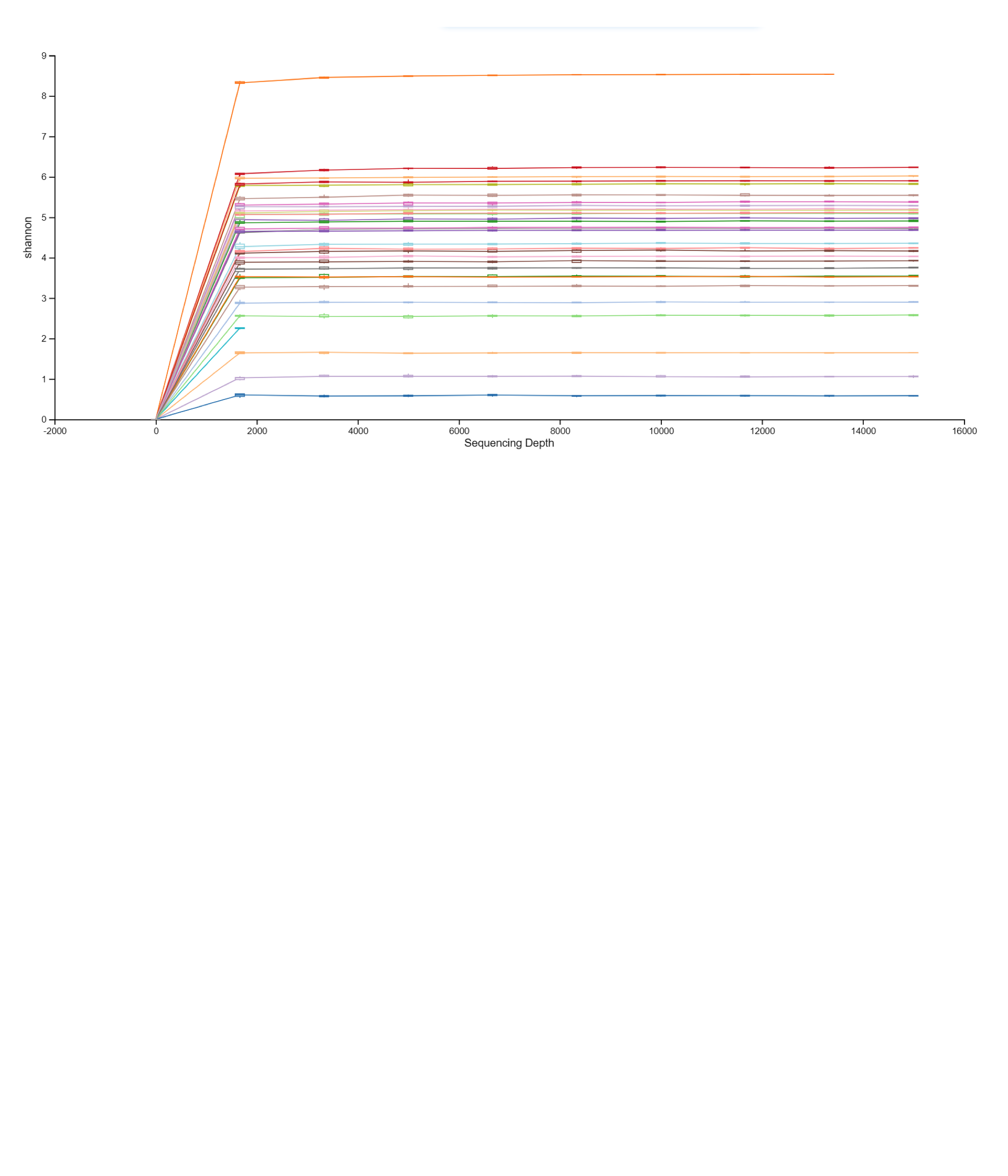

Supplement: S1 Fig — (TIF) [file pone.0243395.s004.tif]

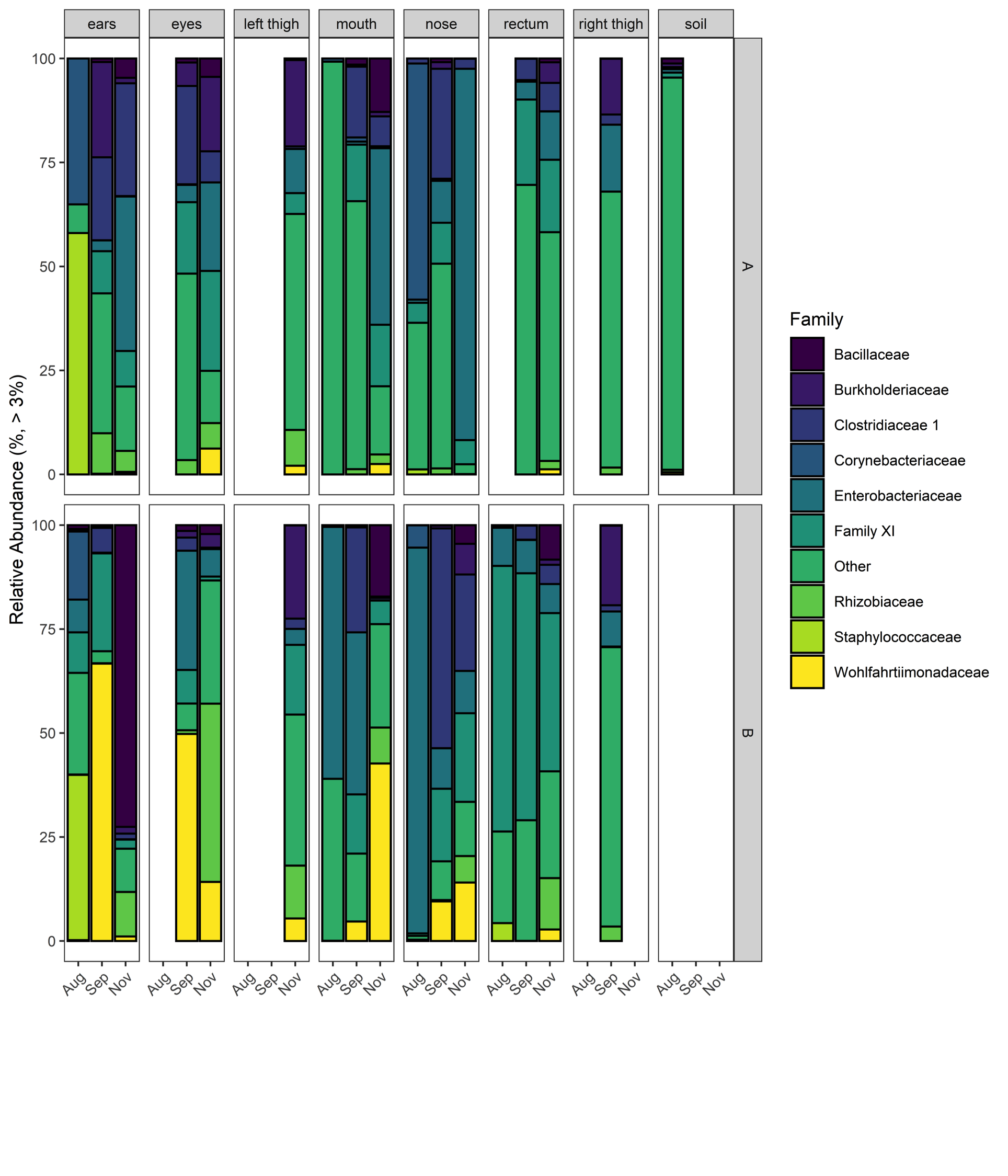

Supplement: S2 Fig — Families shown were represented by at least 3% among samples. (TIF) [file pone.0243395.s005.tif]

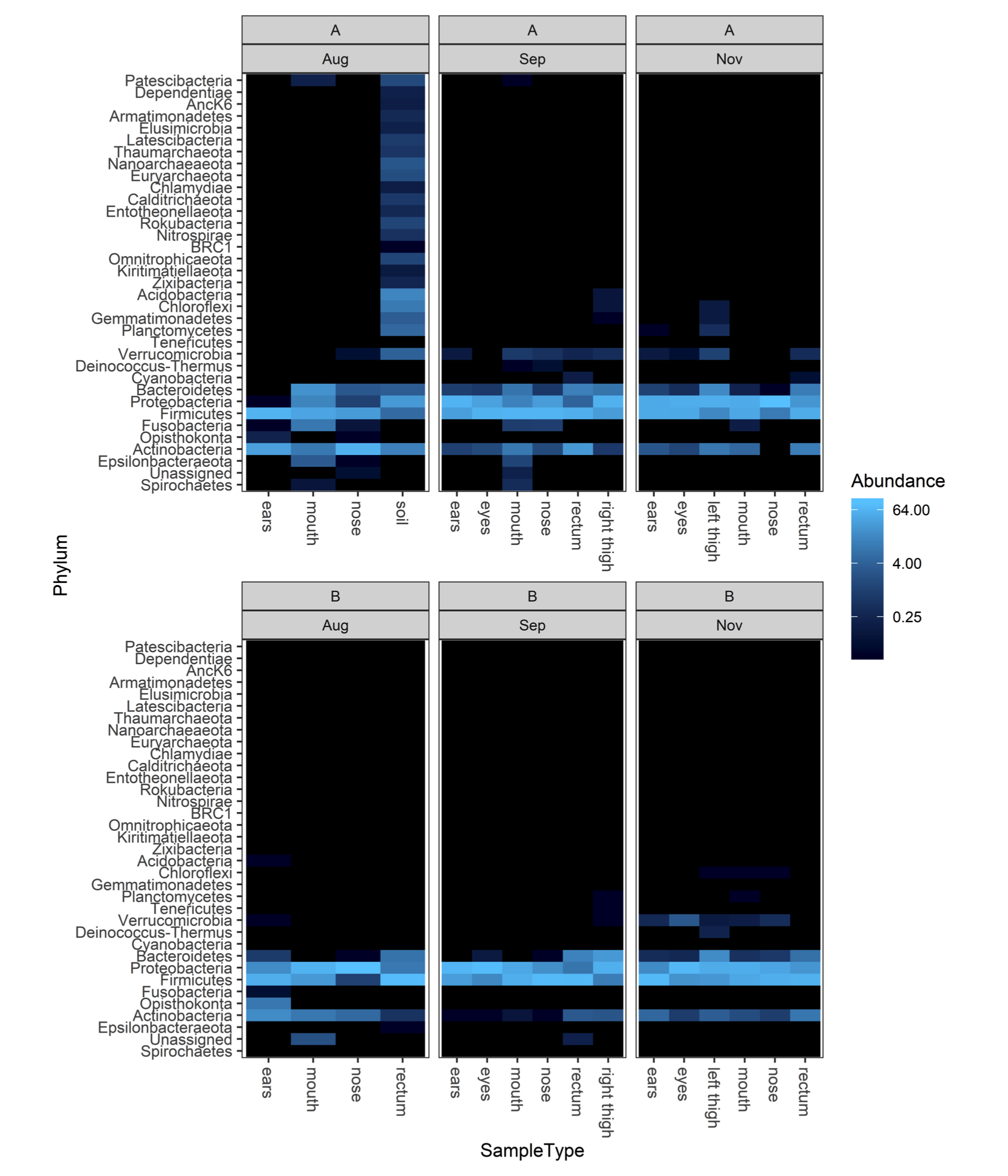

Supplement: S3 Fig — Phyla shown were represented by at least 0.3% among samples. (TIF) [file pone.0243395.s006.tif]

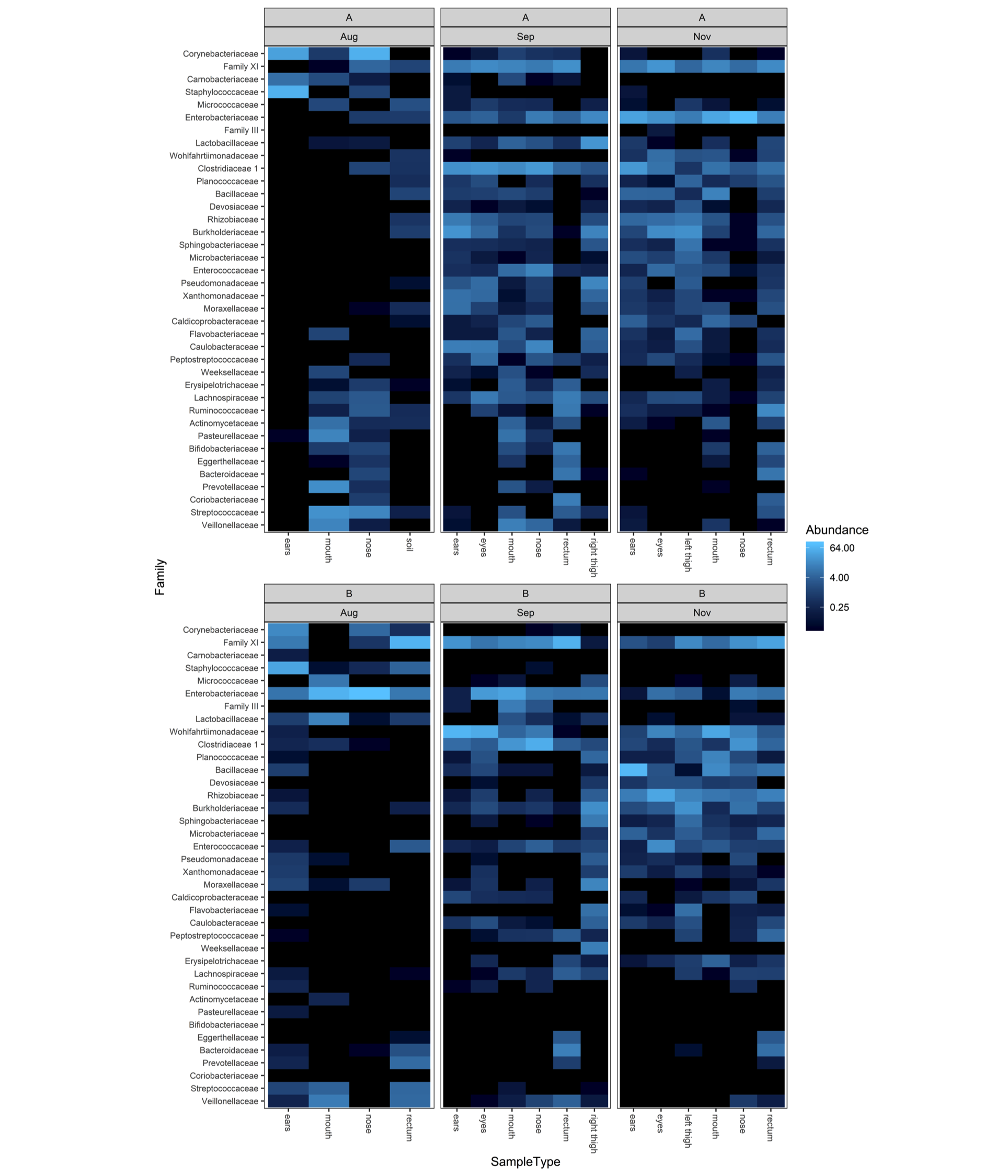

Supplement: S4 Fig — Families shown were represented by at least 0.3% among samples. (TIF) [file pone.0243395.s007.tif]

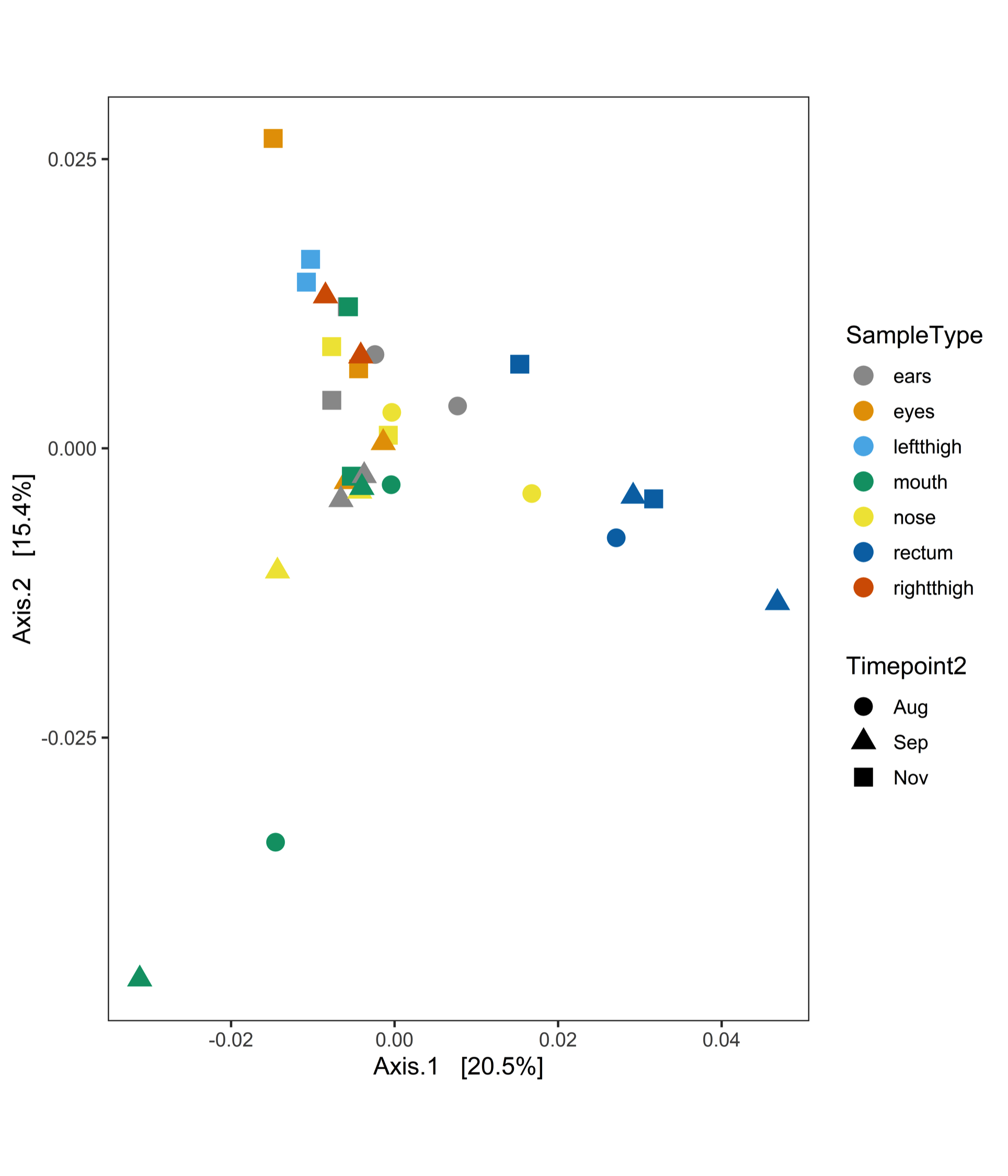

Supplement: S5 Fig — (TIF) [file pone.0243395.s008.tif]

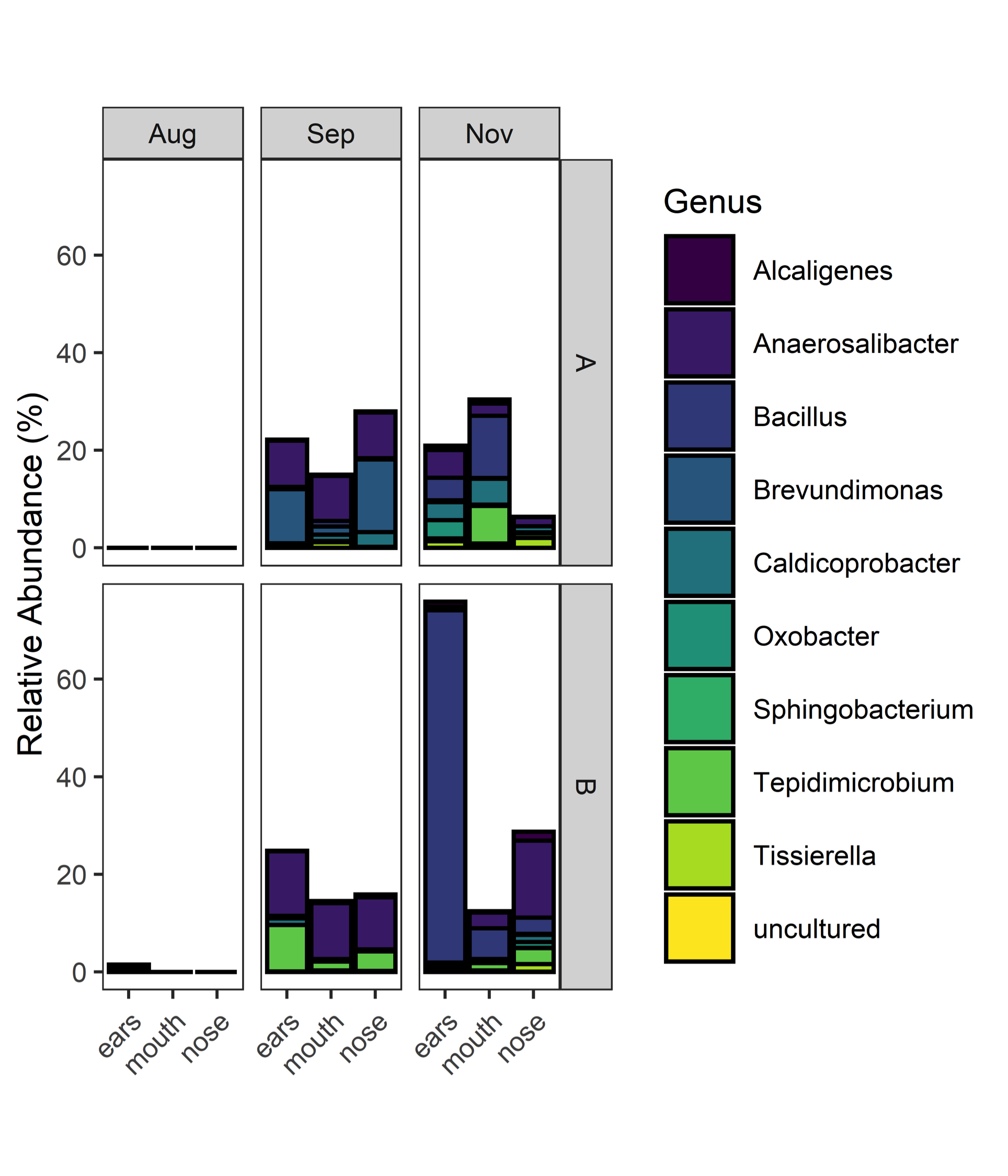

Supplement: S6 Fig — (TIF) [file pone.0243395.s009.tif]
